# A comparative genome-wide study of ncRNAs in trypanosomatids

**DOI:** 10.1186/1471-2164-11-615

**Published:** 2010-11-04

**Authors:** Tirza Doniger, Rodolfo Katz, Chaim Wachtel, Shulamit Michaeli, Ron Unger

**Affiliations:** 1 The Mina and Everard Goodman Faculty of Life Sciences, Bar-Ilan University, Ramat-Gan 52900, Israel; 2 Advanced Materials and Nanotechnology Institute, Bar-Ilan University, Ramat-Gan 52900, Israel

## Abstract

**Background:**

Recent studies have provided extensive evidence for multitudes of non-coding RNA (ncRNA) transcripts in a wide range of eukaryotic genomes. ncRNAs are emerging as key players in multiple layers of cellular regulation. With the availability of many whole genome sequences, comparative analysis has become a powerful tool to identify ncRNA molecules. In this study, we performed a systematic genome-wide in silico screen to search for novel small ncRNAs in the genome of *Trypanosoma brucei *using techniques of comparative genomics.

**Results:**

In this study, we identified by comparative genomics, and validated by experimental analysis several novel ncRNAs that are conserved across multiple trypanosomatid genomes. When tested on known ncRNAs, our procedure was capable of finding almost half of the known repertoire through homology over six genomes, and about two-thirds of the known sequences were found in at least four genomes. After filtering, 72 conserved unannotated sequences in at least four genomes were found, 29 of which, ranging in size from 30 to 392 nts, were conserved in all six genomes. Fifty of the 72 candidates in the final set were chosen for experimental validation. Eighteen of the 50 (36%) were shown to be expressed, and for 11 of them a distinct expression product was detected, suggesting that they are short ncRNAs. Using functional experimental assays, five of the candidates were shown to be novel H/ACA and C/D snoRNAs; these included three sequences that appear as singletons in the genome, unlike previously identified snoRNA molecules that are found in clusters. The other candidates appear to be novel ncRNA molecules, and their function is, as yet, unknown.

**Conclusions:**

Using comparative genomic techniques, we predicted 72 sequences as ncRNA candidates in *T. brucei*. The expression of 50 candidates was tested in laboratory experiments. This resulted in the discovery of 11 novel short ncRNAs in procyclic stage *T. brucei*, which have homologues in the other trypansomatids. A few of these molecules are snoRNAs, but most of them are novel ncRNA molecules. Based on this study, our analysis suggests that the total number of ncRNAs in trypanosomatids is in the range of several hundred.

## Background

Non-coding RNA (ncRNA) genes produce functional RNA molecules, but these molecules do not encode for protein products; rather, these RNA molecules directly participate in various cellular processes. For many years, only a few such ncRNA molecules were known, mainly represented by transfer-RNA (tRNA), ribosomal-RNA (rRNA), small nuclear RNA (snRNA) and small nucleolar RNA (snoRNA). The possible existence of additional types of ncRNA molecules was given little consideration, as the fundamental biological principle was that almost all genes are translated into proteins. As a result, most studies have focused their efforts primarily on protein discovery. The appreciation for the role of untranslated RNAs in the cell has changed dramatically over the past decade. Recent work has shown that the incidence and importance of ncRNA molecules has been underestimated [1-3]. ncRNAs are emerging as key players in multiple layers of cellular regulation [4-7]. In addition, it has been speculated that there are many additional types of ncRNA that have yet to be discovered.

However, systematic computational and experimental identification of these molecules has been difficult. The challenge of predicting ncRNAs from primary sequence is that they lack the known signals, such as start and stop codons as well as the triplet periodicity, which are distinguishing features of protein coding genes. Furthermore, discriminating between ncRNAs and protein-coding mRNAs is not a trivial task. ncRNAs, especially long ones, may contain open reading frames [8,9].

Over the years, several tools for identifying specific ncRNA family members have been developed. These programs generally exploit the fact that some ncRNA classes have relatively well-defined sequence and/or structural characteristics (i.e. tRNAs [10], snoRNAs (H/ACA [11-14] and C/D [15]) and miRNA [16,17]). General non-family specific tools for identifying ncRNA genes have had more limited success. Many ncRNAs have conserved secondary structures, despite having primary sequences that are often highly variable. This resulted in compensatory changes during evolution that are consistent with the conservation of a consensus secondary structure, and can be detected by a stochastic context-free grammar (SCFG) or hidden Markov models (HMMs) that may be used in conjunction with thermodynamic stability (i.e. qRNA [18], RNAz [19]).

ncRNA molecules can be experimentally detected by selecting for small molecules and preparing a cDNA library as was demonstrated by [20]. Most recently, the next generation sequencing technologies have become powerful tools for ncRNA discovery (see [21]). However, laboratory techniques for identifying RNA molecules are often expensive, time-consuming, and labor-intensive. In addition, these experimental methods have a bias toward highly abundant molecules and can miss RNAs that are only present under specific physiological conditions or during specific developmental stages. Thus, *in silico *methods for identifying RNA molecules have greatly complemented experimental work [22-24].

With the availability of many whole genome sequences, comparative analysis has become a powerful tool to study sequence similarities and differences between various organisms. Comparative genomics is an approach that has been used to aid in the discovery of genes, regulatory elements and gene structure [25-27]. It has also been shown as a powerful tool for identifying ncRNA [28-32].

Comparative genomics can serve as a powerful filter for ncRNA; it sifts genomic DNA and yields a subset of sequences that are enriched for ncRNA sequences. Comparative genome-wide studies for the purpose of detecting ncRNAs have been performed in a range of organisms from bacteria to humans. The number of predicted ncRNAs across the evolutionary scale varies widely. In human and higher vertebrates, computational [33,34] and experimental studies [35,36] indicate a number of putative ncRNAs in the range of tens of thousands. In contrast, in urochordates [37], nematodes [38], and drosophilids [39] the predicted numbers are lower, in the range of several thousand. Lower eukaryotes, such as yeast [29], and *Plasmodium *[40,41] are predicted to have ncRNAs in the range of several hundred. Studies of ncRNAs in prokaryotes, such as *E. coli *and other bacteria [18,24,42,43], suggest that the number of ncRNAs is in the low hundreds.

Trypanosomes are unicellular parasites, and are the cause of several devastating diseases affecting humans (e.g. Chagas disease and African sleeping sickness). Trypanosomatids are known for their non-conventional gene expression mechanisms, including RNA editing [44], and *trans*-splicing, a process that is required for the maturation of all mRNAs in these organisms whereby a small exon, encoded by a small RNA, the SL RNA, is donated to all pre-mRNA [45,46]. Trypanosomes have also been used as model organisms to study ncRNA, and over the years the U snRNAs [46], 7SL RNA [47] and snoRNAs [48-52] were described. However, many ncRNAs that have been found in other eukaryotes have not been identified in trypanosomes, such as many snoRNAs involved in RNA processing, RNase P, and telomerase RNA. These molecules remain elusive despite the fact that computer programs (i.e. Snoscan [15]) exist that are specifically designed to search for some classes of ncRNA (i.e. C/D), and are appropriate for identifying trypanosome homologues in genome-scale searches [51]. Based on experimental data from mapping of ribose methylation sites on ribosomal RNA in *T. brucei*, many C/D molecules that guide those modifications still remain to be discovered [49]. Many of the undiscovered ncRNA may have weak or novel motifs that would be impossible to identify without the use of comparative genomics. There have been several *in silico *genome-wide studies in trypanosomes to search for snoRNAs [14,51,53]. Recently, a genome-wide computational study of functional RNA elements in *T. brucei *[54] was published. The genomes of *T. brucei *and *L. braziliensis *were compared using a binomial-based model to assess conservation followed by a QRNA [18] analysis. After filtering by QRNA score and annotation, a total of 53 ncRNA candidates were reported.

Here, we describe a systematic *in silico *screen to identify conserved non-protein-coding genes across multiple trypanosomatid genomes, and prediction of 72 sequences as novel ncRNA candidates. The expression of 50 candidates was tested in laboratory experiments; 18 molecules were shown to be expressed, and for 11 of them there is strong evidence that they represent novel short ncRNAs in procyclic stage *T. brucei*, or their homologues in the other trypansomatids. The RNAs that do not belong to the previously described most abundant families of small RNAs, such as C/D and H/ACA snoRNAs or RNAs binding the Sm or Lsm proteins, were termed RNAs of Unknown Function (RUFs).

## Results

**Figure 1:**
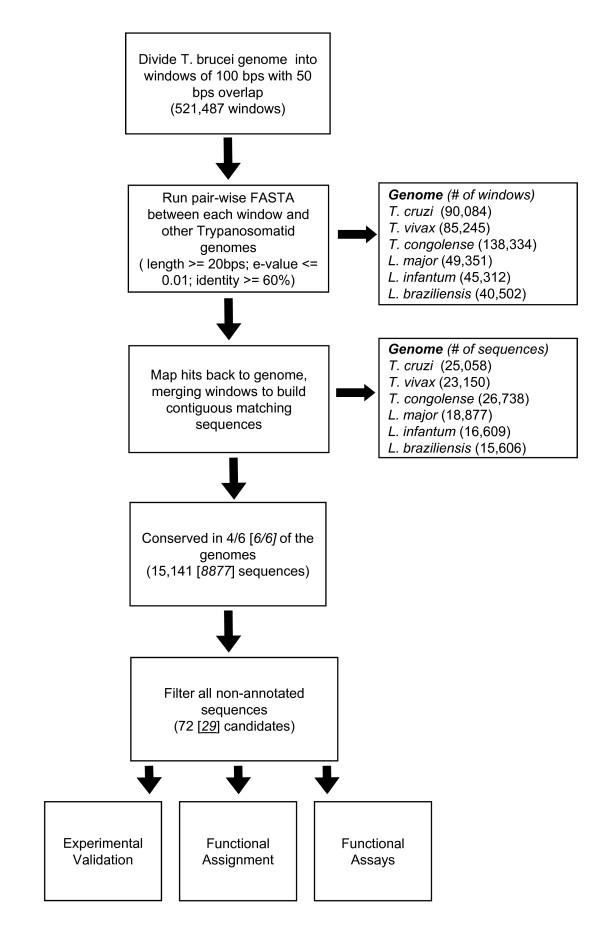
**Schematic representation of the genome wide search pipeline**. Using *T. brucei *as the reference genome, the chart describes each stage, and the number of candidates found at each stage. In the first stage, the number in the parentheses represents the number of homologous windows found. In the following stage, the number in parentheses represents the number of sequences found. The number of sequences conserved in six out of six genomes is given in the square brackets, following the number of sequences conserved in four out of six genomes.

We report here the identification of novel ncRNAs based on the conservation among seven trypanosomatid species. Figure 1 shows the flow of the genome wide ncRNA search pipeline using *T. brucei *as the reference genome. As detailed in the methods, the pipeline is made up of five stages. We began our search with the *T. brucei *genome divided into windows of 100 nts with a 50 nt overlap in between windows, and performed a FASTA search against each one of the six other trypanosomatid genomes. Figure 1 shows the parameters used and the number of results obtained for each stage.

### Assessment of performance

To assess the performance of our prediction scheme, we tested the protocol on the set of known ncRNA molecules of *T. brucei *(GeneDB version 4). When we required conservation in all of the six genomes, we were able to recover almost half of the known ncRNAs. When we loosened our constraints and required conservation in at least four of the six genomes, we were able to return almost 2/3 of the known ncRNAs (Table 1). The threshold of four genomes was chosen, as three of the genomes were from the *Leishmania *genus and three were from the *Trypanosoma *genus; thus conservation over at least four of the six genomes would force the conservation to bridge the divergence between *Leishmania *and *Trypanosoma*. A list of the 559 annotated ncRNA in GeneDB v4 is given in Additional File 1.

**Table 1:** Assessment of performance on known ncRNA found in GeneDB

Type of ncRNA	Annotated in Genedb v4	All 6 genomes	4 of 6 genomes
**rRNA**	106	22 (21%)	92 (87%)

**snRNA**	6	3 (50%)	5 (83%)

**snoRNA**	353	110 (31%)	188 (53%)

**tRNA**	65	64 (98%)	65 (100%)

**misc RNA**	29	28 (97%)	29 (100%)

**Total**	559	227 (41%)	379 (68%)

Using the set of known ncRNA as the standard to evaluate the performance of our algorithm in detecting ncRNAs, the numbers of ncRNA molecules detected are listed, according to families, including those conserved in all six genomes or in 4/6 genomes (note that 6/6 conservation is a subset of the 4/6 conservation). The percent of the ncRNA family that was detected in the screen is listed in the parentheses.

During the analysis, all known and hypothetical protein coding genes were filtered by comparing the coordinates of the candidate sequences to those of the annotation. This filter left a pool of 125 potential ncRNA candidates that are conserved in a minimum of four of the six genomes. However, the initial filtering of annotated sequences was based on comparing the coordinates of the sequences as appears in GeneDB. This comparison is fast, but it may miss proteins because of coordinate annotation problems, which are quite common. Thus, we checked the 125 candidates further by direct sequence comparison to see if they match any annotated gene. As annotation in the *T. brucei *genome is incomplete, we compared our candidates against the annotated genes of both *T. brucei *and *L. major*. Using BLAST comparison versus the *T. brucei *and *L. major *annotated sequences, a significant number of candidates (47 of the 125) were found to be highly similar to known coding sequences. Most of these sequences were simply a result of incomplete genome annotation. For example, several of the ribosomal RNA proteins (LmjF28.2460 ribosomal protein S29, putative and LmjF36.3750 40S ribosomal protein S27), which are highly conserved, were not annotated in *T. brucei*. We also found six previously described RNAs that are not reported in GeneDB. For example, the screen identified selenocysteine-tRNA [55], whose sequence had been unannotated in the genome, while instead sRNA-76 [56] was labeled as selenocysteine-tRNA, and was also identified in the final set (candidate 7). A list of the additional RNA genes that have been reported previously in the literature, but have not yet been incorporated or are misannotated in the GeneDB genome annotation is provided as Supplementary Material (Additional File 2). These include MRP RNA [49], snR30 [48], U5 [57], tRNA-sec [55], sRNA-76 [56], and several previously identified snoRNA clusters [14,49,51].

At this point we were left with a total of 72 candidates that are conserved in 4/6 genomes, out of which 29 are conserved in 6/6 genomes. Table 2 summarizes the number of sequences found in the 6/6 and 4/6 genome conservation analysis categorized according to their annotation. The complete list of all the sequences of the 72 ncRNA candidates is provided as Additional File 3. Searches of the RFAM database using BLAST on these 72 sequences did not provide any additional annotation information, suggesting that these may be trypanosome specific ncRNAs, or alternatively the sequence similarity to other organisms is too low to be detected. Note that our method cannot detect the strand that contains the candidate molecule as conservation is the same for both strands. However, since trypanosomes have polycistronic transcription, we can obtain information about the direction of transcription from that of flanking genes. In cases where flanking genes were not sufficient to determine the direction of transcription, the sequences from both strands were subjected to the experimental validation step described below.

**Table 2:** Number of candidates from the different subtypes of RNA

	4 of 6 genomes	All 6 genomes
**Total**	15141	8877

**Annotated proteins**	7871	5482

**Hypothetical proteins**	6819	3139

**Known ncRNA**	379	227

**Not annotated**	72	29

Total loci found when requiring conservation in all six genomes or when requiring at least four genomes. The total was then further categorized based on each loci's annotation.

We checked for redundancy between the 72 candidates and found that Candidates # 85 and #90 shared 98% identity to each other and 70% identity with candidate #78. Candidates # 89 and #99 shared almost 100% identity to each other and 88% identity with candidate # 124. Candidates 68 and 70 shared 63% identity. Interestingly none of these candidates were among the molecules that we were able to validate experimentally.

### Experimental Verification

Fifty of the 72 candidates in the final set were chosen for experimental validation. Fifteen were chosen from the sequences that were conserved in six of six genomes, and the rest were chosen randomly from the remaining candidates. The list of candidates sent for experimental verification appears in the comments to Additional File 3, and Additional File 4 provides the list of primers. Eighteen of the 50 candidates were shown to be expressed in cells. Expression was detected by primer extension assay that exactly determines the 5' end of the molecule. The strength of the signal reflected the abundance of the RNA, as the same amount of radio labelled primer and RNA were used in each experiment. Note that we did not use an internal control of very abundant RNA because it often affects the ability of non-abundant RNA to prime. Rather, we performed the primer extension using U3 snRNA as an internal control. This RNA was chosen because it is stable and tends not to degrade. However, the presence of the U3 oligo in the reaction reduced the efficiency of extension from our tested RNA (see Additional File 5).

**Figure 2:**
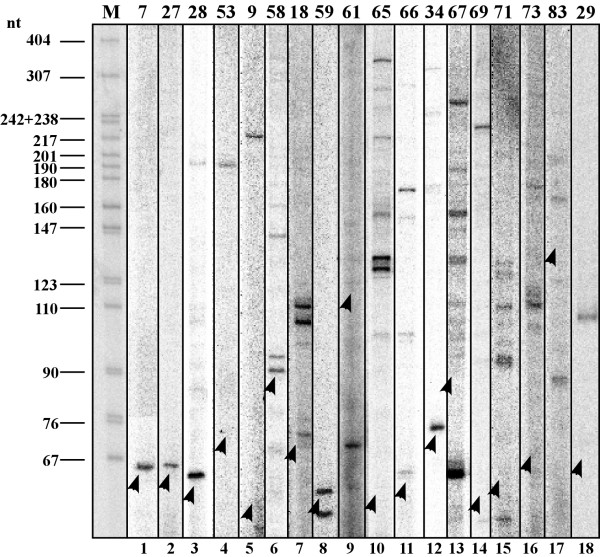
**Results of expressed candidates in primer extension assay**. RNA was subjected to primer extension using the oligonucleotide specified in Additional File 4. The products were separated on a 6% polyacrylamide. M-DNA marker, labelled *pBR322 *DNA *Msp*I digest. The arrowheads indicate the 5' end of full length transcripts. The numbers above the lanes indicate the candidates listed by candidate number as found in column 1 in Additional File 3, while the numbers below the lanes are sequential. Note that the gel is a composite. However, each experiment was performed in the same way. The same batch of total RNA was used, and the same amount of gel purified primer was used (50,000 cpm). Of the lanes analyzed, 11 (1,2,3,4,5,7,8,11,12,14,18) show extension products that are distinct or highly dominant and agree with the size predicted by the bioinformatic analysis.

Out of the 50 molecules, 32 did not show expression in the primer extension experiment described above. 18 molecules did yield extension products and 11 of the 18 had a distinct extension product suggesting that they are distinct small RNAs. The others yielded multiple bands, which may reflect the extension of a long polycistronic RNA, but probably not of a single small RNA (see Figure 2). While we mapped the 5' end of the candidates by primer extension, the full size of the products is unknown, as there is no information about their 3' end. However, for most of the distinct bands (and for some of the multiple bands) the size predicted by the bioinformatic analysis was quite reliable. This is a surprising and encouraging result considering the thresholds and cut-offs that are inherently somewhat arbitrary in bioinformatic analysis.

Note that even some of the candidates that were not expressed may still be ncRNAs that are expressed in another part of the parasite's life cycle. We analyzed expression only in procyclic form, and it is possible that the other RNAs are stage specific and are expressed only at 37°C when the parasite lives in the mammalian host. Indeed, we previously identified snoRNAs that are expressed better in the bloodstream form [49]. However, for the purposes of evaluating the performance of our procedure, we considered candidates that did not show a distinct band in our assay as false predictions.

Although Northern blot would be a better approach to show that the identified candidates are indeed small RNAs, the majority of the novel RNAs identified by this study were not abundant. There are only two that were abundant as determined by primer extension: tRNA-sec and candidate #28. tRNA-sec does appear abundant in the Northern blot, but candidate #28, while appearing strong by primer extension, gives a relatively weak band by Northern analysis (see Additional File 6); hence the remaining molecules, which were not abundant on the primer extension assay, are not likely to be clearly detected by Northern analysis. Note that in these two cases where we compared primer extension with Northern analysis, the sizes of the molecules were consistent.

In order to evaluate the performance of our prediction scheme we needed to estimate the True Positive (TP), True Negative (TN), False Positive (FP) and False Negative (FN) rates of the ncRNA prediction. TP represents the predictions that turn out to be correct, and our analysis yielded 379 (the number of known ncRNA molecules that we "identified") plus the 11 molecules we confirmed experimentally. FN can be estimated by the number of known ncRNA that our methods missed and there are 180 such molecules (559 known ncRNA molecules minus the 379 detected). FP corresponds to the number of predictions that were shown to be wrong which is 39 (50-11). Calculating the TN values is meaningless since most of the genome is not comprised of ncRNA. Thus, the calculated TN value would be in the millions, and while this would make the performance measures that are dependent on TN (like Specificity which is defined as TN/(TN+FP)) seem to be extremely good, this doesn't reflect true performance characteristics.

However, even when ignoring TN, we can estimate the Sensitivity (defined as TP/(TP+FN) to be 0.68 and the Positive Predictive Value (PPV also known as Precision, defined by TP/(TP+FP)) to be 0.9. Note that if we consider the additional 18 molecules that show expression (although with multiple bands) as positive predictions, as well, the score would be even somewhat higher.

To examine if the novel RNAs belong to known families of RNA, we examined their level in *T. brucei *cells depleted of core RNA proteins by RNAi-silencing. NOP58 silenced cells (previously described [49]) were used to classify RNAs as C/D snoRNAs, and CBF5 silenced cells [48] were used to identify H/ACA RNAs. Five of the identified RNA species were assigned to their respective families (4 C/Ds, and 1 H/ACA), and the others remain RNAs of unknown function (RUFs). The level of the RUFs was examined in cells silenced for the C/D and H/ACA core proteins as described above, and in cells depleted for Lsm8 and SmD1, and their levels were unchanged, suggesting that these are novel small RNAs, not belonging to known classes of small RNAs, and have binding proteins that are yet to be discovered.

### Novel snoRNAs

**Figure 3:**
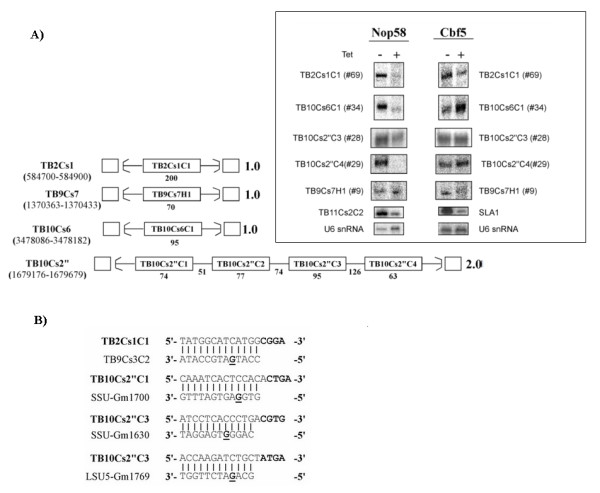
**Effects of NOP58 and CBF5 knockdown**. (a) Effects of NOP58 and CBF5 silencing on novel snoRNAs and schematic representation of the clusters encoding the snoRNAs. RNA was prepared from cells carrying either NOP58 or CBF5 silencing constructs before induction with Tetracycline (-), and 3 days after addition of tetracycline (+). The RNA was analyzed by primer extension and separated on a 6% denaturing polyacrylamide gel. The level of U6 snRNA was used to examine the amount of RNA the samples. The same RNA was used for the different primer extension assays. The positions of the snoRNAs in the genome are given on the left. The sizes of the snoRNAs and the intergenic regions (in nucleotides) are given below each diagram. The number of times the gene cluster (delineated by parentheses) is repeated is given on the right. The inset shows the controls: TB11Cs2C2, a known C/D molecule, and SLA1, a known H/ACA molecule, for which a significant reduction in expression following silencing can be seen, and U6 snRNA, a non-snoRNA molecule which is not affected. Novel C/D and H/ACA molecules were named as such based on the nomenclature of Liang et al. 2005 [49]. (b) Putative interaction domains for the C/D molecules TB2Cs1C1, TB10Cs2''C1, and TB10Cs2"C3. For TB10Cs2''C3, two putative targets are listed.

Most eukaryotic C/D box and H/ACA snoRNAs guide 2'-O methylation (Nm) and pseudouridylation on specific nucleotides on the rRNA or snRNAs, and are also involved in rRNA processing [5]. To date, 64 C/D snoRNAs and 48 H/ACA snoRNAs [14,49-51] have been described in *T. brucei*, and 62 C/D and 37 H/ACA snoRNAs [53] were described in *L. major*. Among the candidates, four C/D box (candidates 28, 29, 34, and 69) and one H/ACA snoRNA (candidate 9) (See Figure 3a for cluster structure and experimental gels) were found. Candidates 28 and 29 were found as a cluster, and upon further inspection of the flanking region, two additional C/D snoRNAs were identified in this cluster. Candidates 9, 34 and 69 were found in the genome as single-copy genes. Proposed interaction domains for several of these snoRNAs are presented in Figure 3b, while no putative target was identified for the others. Interestingly, a continuous 13 bp complementarity was identified between TB2Cs1C1 and another C/D snoRNA TB9Cs3C2 [51]. The box structure of the four C/D snoRNA presented in Figure 3a is depicted in Additional File 7. Positive and negative controls for these experiments are included as Additional File 8.

### Novel RNA Candidates of unknown Function (RUFs)

**Figure 4:**
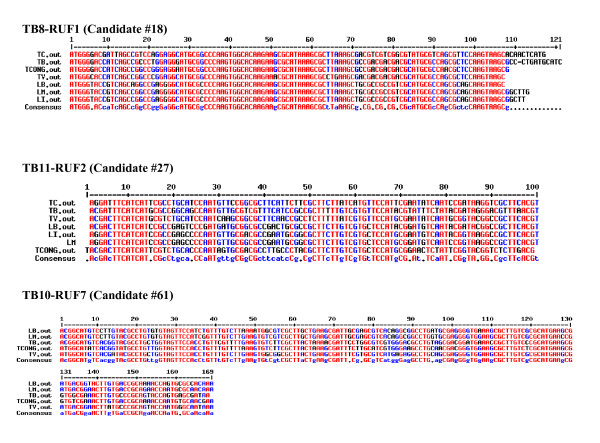
**Examples of multiple sequence alignments of RUFs among different trypanosomatid species**. Several examples illustrating the high level of conservation of the RUFs: These multiple sequence alignments were produced using the MulitAlign server [79]. High consensus (> 90% of the column is conserved) is indicated in red. Low consensus is indicated in blue (> = 50% conservation). Neutral columns are indicated in black.

The remaining candidates were not readily identifiable as belonging to any of the known ncRNA families. These sequences were highly conserved across multiple trypansomatids, and were not found in open reading frames. Several examples of multiple sequence alignments depicting the high conservation of these RUFs among different trypanosomatid species are shown in Figure 4. In addition, two candidates show potential base-pair complementarity to areas on ribosomal RNA. TB11-RUF5 has potential perfect complementarity to 13 continuous base pairs on LSU-β (296-308), and TB11-RUF2 has potential perfect complementarity to 12 continuous base pairs on LSU-β (337-348). Other candidates have potential complementarity to additional areas in the genome. TB8-RUF1 has potential complementarity of 19 out of 20 residues to a known coding sequence Tb927.8.1590/Tb08.29O9.320 (upl3 ubiquitin-protein ligase), and perfect 15 base-pair complementarity to Tb927.7.2080/Tb07.43M14.530 (methyltransferase, putative). TB7-RUF8 has potential perfect complementarity to 16 continuous base pairs of Tb10.70.5440 (chaperone protein DNAJ, putative). The biological significance of this finding is currently unknown, since the statistical significance of complementarity with a run of even 15-20 nucleotides is not high when the entire genome is scanned. However, the target genes mentioned above are key regulators of proteolysis, chromatin state and protein folding, and these putative RUFs may function in regulating their level. This will require further experimental validation.

## Discussion

We divide the Discussion into two sections; the first section deals with the technical aspects of the comparative genomics procedure, while the second will describe the implications of our findings on the repertoire of ncRNA molecules in Trypanosomes.

### FASTA versus BLAST for RNA comparative genomics

For the purpose of RNA comparative genomics, one has to choose the most appropriate tool to efficiently compare the genomes with optimal sensitivity for detecting homologous ncRNA. BLAST [58] and FASTA [59] are the two popular heuristic programs for searching query sequences against a sequence database. Several papers have been published benchmarking the performance of BLAST and FASTA in protein-coding similarity searches [60,61]. One study [62] evaluated the sensitivity and specificity for the detection of ncRNA based on a variety of homology methods including BLAST and FASTA. Overall, FASTA was found to be more sensitive in detecting ncRNA than BLAST. In addition, FASTA's performance in detecting ncRNA was found to be comparable to WU-BLAST [63], though FASTA's run- time was faster. Nonetheless in the ncRNA community at large, the most popular tool of choice has been and continues to be BLASTN (i.e. [12,30,39,42,64]). As a test case for the preferential homology search methodology, the detection of a known snoRNA cluster (LM25Cs1) in *L. major *was examined. BLASTn and FASTA searches were performed using as the query a 100 kb area in *L. major *which included the snoRNA cluster, versus the whole *T. brucei *genome as the database. Based on our results from this small sample, FASTA, using the default settings, is more sensitive at identifying ncRNA even when we used more sensitive parameters for BLASTN (-r 1, -q -1 instead of the default +1/-3, personal communication William Pearson). We also tested the sensitivity of performing the sequence comparison programs on the whole 100 kb, and on windows of 100 bps with 20% and 50% overlap. The result of these experiments, which is consistent with [62], is that FASTA should be preferred over BLAST for ncRNA searches.

### Implications for the repertoire of ncRNA in Trypanosomes

In this study, a systematic *in silico *screen for conserved ncRNA among seven trypanosomatids is presented. In total, we found close to 100 candidates. One reason for the relatively low number of the additional ncRNAs that we found stems at least in part from the fact that studies from our labs, and those of others (i.e. [47,49,56,57,65,66]), already characterized the repertoire of Trypanosome snoRNAs, snRNAs, and other ncRNA species. Many of the recent studies which utilized comparative genomics to identify ncRNAs examined organisms that had very little previous ncRNA annotation. For example, in a study of *Plasmodium*, Chakrabarti et al. [40] identified several snRNAs (U1-U5), telomerase RNA, and about 30 snoRNAs. We believe that the fact that we were able to detect a third to half of the known ncRNA in trypanosomes by the bioinformatic method used indicates that our computational procedure is thorough.

In a recent study, Mao et al. [54] evaluated the conservation between *T. brucei *and *L. braziliensis *using a binomial-based model. QRNA was then used to identify likely ncRNA candidates. A total of 378 sequences were found with a significant QRNA score. Among the 378 sequences, 117 sequences were found to be highly significant when compared to randomized versions of the same sequence. Of the 117, 53 were unannotated. We evaluated the overlap between our final set and Mao's set of 378. We found three common sequences. They were: VSG pseudogene (candidate #121), a retro-transposon hotspot (candidate #98), and a novel C/D snoRNA (candidate #29, named TB10Cs2"C4). Note that although Mao et al. reported a low false positive rate - their algorithm only detected about 50% of the tRNAs, 20% of the rRNAs and 0% of the known snoRNAs. Comparing the performance of our procedure with this work, we conclude that the procedure used in our study is efficient and can serve as a useful tool for other systems, as well.

We propose that our findings can be used to estimate the total number of small ncRNA molecules in Trypanosomes. Of the 50 candidates tested, 18 novel ncRNAs were validated in procyclic stage trypanosomes. The experimental validation of a sample of 50 candidates suggest that about 1/3 of the candidates exist as novel small RNAs. On the other hand, when we tested our procedure on the known ncRNA we found that about 2/3 of the molecules have sufficient sequence conservation to be discovered by comparative sequence methods. Assuming that the rest of the ncRNA repertoire has similar characteristics and combining the two observations above, we can suggest that the total number of ncRNA molecules yet to be discovered in trypanosomatids is unlikely to be more than a few hundred.

There are several caveats to this claim. First, in our search, we did not consider the large amount (about 60% of the genes) of conserved hypothetical proteins. Many hypothetical proteins have been annotated as such because their sequence is found in open reading frames. However, some of these sequences may actually harbor ncRNA molecules. Several snoRNAs have been found within open reading frames. For example, Tb03.30p12.690, labelled as a hypothetical protein, overlaps with a C/D snoRNA TB3Cs2C1.

In addition, it is possible that there are many ncRNAs that are organism specific and cannot be detected by comparative methods. We notice that our study failed to identify several RNAs that are expected to exist in trypanosomatids such as telomerase RNA and RNAse P. Interestingly, Piccinelli et al. [67] studied RNase P and MRP in a variety of eukaryotes, but were unable to identify them in trypanosomatids. This is likely due to the fact that these RNAs are highly divergent even among closely related trypanosomatids. An interesting finding in this context is the detection of snoRNAs (TB2Cs1C1, TB10Cs6C1 and TB9Cs7H1) that are present in the genome as singletons, and are not part of the usual cluster organization of snoRNA in trypanosomatids. While obviously these two molecules were conserved enough among the different trypanosomatid species to be detected, other singleton molecules may be more diverse and hence harder to detect, suggesting that more such snoRNAs may exist.

Third, our extrapolation was based on our observation that only about 1/3 of candidate molecules were shown to be expressed. We cannot rule out the possibility that these candidate molecules are expressed at different stages in the life cycle of the parasite or under ambient environmental conditions. In *C. elegans *[68], it was shown that many ncRNAs are developmentally regulated and exhibit stage-specific function.

## Conclusion

Taken together these issues limit our ability to quantitatively estimate, the number of ncRNA molecules in trypanosomatids. However, even if each one of these factors are off by a factor of two, our overall estimate should be in error by less than a single order of magnitude. Thus, we believe that our results supply an "order of magnitude" qualitative argument suggesting that there are relatively few remaining small ncRNA to be identified. Since we found several dozen candidates, we estimate that not more than several hundred ncRNA molecules exist in each of the trypanosomatid genomes. Many of these molecules may be additional members of known ncRNA families, so that the expected number of novel families is limited.

It has been suggested that the genome of higher eukaryotes contain many thousands of as yet undiscovered ncRNA molecules. Washietl et al., [19], suggested that this repertoire includes short and long ncRNA molecules. Indeed, there is mounting evidence [69] that there are thousands of long ncRNA molecules (although their functional relevance is still under debate). However, we must note that there is no experimental evidence to support the claim of a large number of short ncRNA, except for the large variety of very short ncRNA (miRNA, piRNA) which are associated with the Dicer/Argonaut silencing system. Our findings support the view that at least for unicellular eukaryotes, the repertoire of small ncRNA is not likely to grow much beyond what is already known, and will remain in the hundreds and not thousands.

## Methods

### Genomic Data sources

*Trypanosoma brucei *(TB) genomic DNA and sequence annotation (version 4) was downloaded from GeneDB (http://www.genedb.org). GeneDB contains all available sequences from the 11 megabase chromsomes of *T. brucei *strain TREU927/4 GUTat10.1 generated by the *T. brucei *genome projects at The Institute for Genomic Research (TIGR's *T. brucei *project) and The Wellcome Trust Sanger Institute (Sanger's *T. brucei *project). *Trypanosoma cruzi *(version 4) (TC), *Leishmania major *(version 5.2) (LM), *Leishmania infantum *(version 2) (LI), and *Leishmania braziliensis *(version 1) (LB) genomic sequence data was also downloaded from GeneDB (ftp://ftp.sanger.ac.uk/pub/pathogens/). The nuclear genome of *Trypanosoma cruzi *CL Brener is being sequenced by the TIGR-Seattle Biomedical Research Institute-Karolinska Institute *T. cruzi *Sequencing Consortium (TSK-TSC) (http://www.jcvi.org/). The genome of *L. major Friedlin*, the reference strain (MHOM/IL/80/Friedlin, zymodeme MON-103), was sequenced as part of a multi-centre collaboration (Sanger Institute/EULEISH, Seattle Biomedical Research Institute, FMRP). The shotgun sequences of *T. vivax *(TV) and *T. congolense *(TCONG) were downloaded from GeneDB (ftp://ftp.sanger.ac.uk/pub/databases/). The Sanger Institute has also carried out a 5× coverage of the nuclear genome of *T. vivax*, as well as *T. congolense*. The sizes of the *T. brucei*, *T. cruzi*, *L. major *genomes are 25 Mb, 60 Mb, and 32 Mb respectively. These genomes have been published [70-72]. The *L. major *and *T. brucei *genomes are fully assembled. The *T. cruzi *genome has been fully sequenced, but its assembly is still in its preliminary stages. The *T. cruzi *genome is available as many large contigs.

### Sequence similarity searches

The basis for our search strategy was to designate one trypanosomatid genome as a "reference" and to find all sequences in the other organisms that are similar to it. We chose to use *T. brucei *as the reference genome since it is fully sequenced, reasonably annotated, and because we have the experimental setup for candidate validation. While genome synteny maybe the preferable method to align genomes, we note that some of our genomes are not assembled and are still only available as shotgun sequences; thus we had to chose an alignment method that is based on relatively small windows. The reference genome, *T. brucei*, was divided into a window size of 100 bps with a sliding window of 50 bps. These sequences were searched for similarity against the other trypanosomatid genomes using FASTA [59]. We found FASTA to be more useful for this project than BLAST (see the Discussion). A Bio-PERL/PERL [73] script was written to post-process the FASTA results. Sequence matches were further analyzed if they fit the following criteria: 25 bps or longer, an e-value less than or equal to 0.01, and percent identity equal or greater than 60%. FASTA matches that passed the filter were then mapped back to the *T*. *brucei *genome. Areas that were less than 10 bps apart were concatenated. Conservation was defined by the number of genomes that had matches to the same corresponding segment of the genome. We considered areas that were conserved in at least four of the six genomes and those that were conserved in all six genomes. Sequences annotated as protein coding or hypothetical protein coding, were then filtered out.

### General ncRNA Detection tools

BLAST [58] was run using the *T. brucei *ncRNA candidates versus the RFAM database (v6.1) [74] to search for sequence similarity to any known ncRNA.

### Experimental Methods

#### Primer extension

RNA was prepared from *T*. *brucei *cells using the TRI-Reagent (Sigma). Primer extension analysis was performed as described [75,76] using 5'-end-labeled oligonucleotides specific to each target RNA. The extension products were analyzed on a 6% polyacrylamide/7 M urea gel and visualized by autoradiography. For examining the level of ncRNAs under silencing of the core RNA binding proteins, RNA was prepared from untreated cells and 3 days after the induction of silencing, as previously described [48,49,77,78].

## Sequence Availability

Sequence data from this study were deposited in GeneDB. Accession numbers can be found in Additional File 9.

## Authors' contributions

TD carried out all the computational work in this study. The experimental work was performed by RK and CW. SM and RU coordinated the project. TD, SM and RU wrote the manuscript. All authors have read and approved the final manuscript.

## Supplementary Material

Additional file 1 **List of the annotated GeneDB v4 RNA genes in *T. brucei***. List of the annotated ncRNA found in GeneDB ver4, which were used as a standard to assess the success of our screen. Click here for file

Additional file 2 **List of missing/mis-annoted ncRNA**. List of the additional RNA genes that have been reported previously in the literature, but have not yet been incorporated or are misannotated in the GeneDB genome annotation. Click here for file

Additional file 3 **Complete list of candidate ncRNA**. The complete list of all the sequences of the 72 ncRNA candidates conserved in four of the six genomes. The first 29 were conserved in all of the six genomes. Click here for file

Additional file 4 **Oligonucleotides used in primer extension for candidates presented in Figure **2. Click here for file

Additional file 5 **Results of expressed candidates in primer extension assay with an internal control**. Primer extension was performed as in Figure 2 with the addition of an internal control to each sample. The primer extension reactions contained a primer specific for the candidate as well as a primer specific to U3 snoRNA. Click here for file

Additional file 6 **Northern blot analysis of two of the candidates**. RNA was prepared from PS cells, separated on a 10% denaturing polyacrylamide gel, and subjected to Northern analysis with the indicated oligonucleotide anti-sense probes. Click here for file

Additional file 7 **The box structure of the novel C/D molecules**. For C/D snoRNA TB10Cs2"C3, TB10Cs2"C4, TB10Cs6C1, TB2Cs1C1 the canonical C and D box structure is shown. Click here for file

Additional file 8 **Effects of NOP58 and CBF5 knockdown on all candidates**. RNA was prepared from cells carrying either NOP58 or CBF5 silencing constructs before induction with Tetracycline (-) and 3 days after addition of tetracycline (+). The RNA was analyzed by primer extension and separated on a 6% denaturing polyacrylamide gel. The level of U6 snRNA was used to examine the amount of RNA the samples. The same RNA was used for the different primer extension assays. Click here for file

Additional file 9 **GeneDB accession numbers of the new snoRNA molecules that were reported in this study**. List of the newly annotated sequences with their GeneDB id. Click here for file
