# Supplementary material for: A comparative genome-wide study of ncRNAs in trypanosomatids
Source: BMC Genomics. 2010 Nov 4;11:615. doi: 10.1186/1471-2164-11-615 (PMC3091756; doi:10.1186/1471-2164-11-615)
Supplement: Additional file 1 — List of the annotated GeneDB v4 RNA genes in T. brucei. List of the annotated ncRNA found in GeneDB ver4, which were used as a standard to assess the success of our screen. [file 1471-2164-11-615-S1.DOC]

| **Name** | **Type** | **Chromosome** | **Start** | **End** | **Strand** | GeneDB ID | **Comments** |
| --- | --- | --- | --- | --- | --- | --- | --- |
| U1 | snRNA | Tb927_08_v4 | 857771 | 857845 | -1 | Tb927.8.2855 |  |
| U2 | snRNA | Tb927_02_v4 | 1024578 | 1024950 | 1 | Tb927.2.5680 |  |
| U3 | snRNA | Tb927_08_v4 | 861696 | 861839 | 1 | Tb927.8.2864 |  |
| U4 | snRNA | Tb927_10_v4 | 1866946 | 1867055 | 1 | Tb10_snRNA_1 |  |
| U5 | snRNA | Tb927_10_v4 | 1906846 | 1906907 | -1 | Tb10_snRNA_2 | Wrong annotation |
| U6 | snRNA | Tb927_04_v4 | 325767 | 325860 | 1 | Tb927.4.1213 |  |
| SL RNA | misc RNA | Tb927_09_v4 | 2236261 | 2236306 | 1 | Tb09_SLRNA_0001 |  |
| SL RNA | misc RNA | Tb927_09_v4 | 2242621 | 2242748 | 1 | Tb09_SLRNA_0002 |  |
| SL RNA | misc RNA | Tb927_09_v4 | 2244013 | 2244146 | 1 | Tb09_SLRNA_0003 |  |
| SL RNA | misc RNA | Tb927_09_v4 | 2245396. | 2245529 | 1 | Tb09_SLRNA_0004 |  |
| SRP RNA, 7SL | misc RNA | Tb927_08_v4 | 860996 | 861256 | -1 | Tb927.8.2861 |  |
| SL RNA | misc RNA | Tb927_09_v4 | 2252390 | 2252523 | 1 | Tb09_SLRNA_0009 |  |
| SL RNA | misc RNA | Tb927_09_v4 | 2250982 | 2251115 | 1 | Tb09_SLRNA_0008 |  |
| SL RNA | misc RNA | Tb927_09_v4 | 2249577 | 2249710 | 1 | Tb09_SLRNA_0007 |  |
| SL RNA | misc RNA | Tb927_09_v4 | 2248181 | 2248314 | 1 | Tb09_SLRNA_0006 |  |
| SL RNA | misc RNA | Tb927_09_v4 | 2246773 | 2246906 | 1 | Tb09_SLRNA_0005 |  |
| SL RNA | misc RNA | Tb927_09_v4 | 2259243 | 2259376 | 1 | Tb09_SLRNA_0014 |  |
| SL RNA | misc RNA | Tb927_09_v4 | 2260621 | 2260754 | 1 | Tb09_SLRNA_0015 |  |
| SL RNA | misc RNA | Tb927_09_v4 | 2256484 | 2256617 | 1 | Tb09_SLRNA_0012 |  |
| SL RNA | misc RNA | Tb927_09_v4 | 2257855 | 2257988 | 1 | Tb09_SLRNA_0013 |  |
| SL RNA | misc RNA | Tb927_09_v4 | 2253752 | 2253885 | 1 | Tb09_SLRNA_0010 |  |
| SL RNA | misc RNA | Tb927_09_v4 | 2255116 | 2255249 | 1 | Tb09_SLRNA_0011 |  |
| SL RNA | misc RNA | Tb927_09_v4 | 2263409 | 22635421 | 1 | Tb09_SLRNA_0017 |  |
| SL RNA | misc RNA | Tb927_09_v4 | 2262015 | 2262148 | 1 | Tb09_SLRNA_0016 |  |
| SL RNA | misc RNA | Tb927_09_v4 | 2266146 | 2266279 | 1 | Tb09_SLRNA_0019 |  |
| SL RNA | misc RNA | Tb927_09_v4 | 2264785 | 2264918 | 1 | Tb09_SLRNA_0018 |  |
| SL RNA | misc RNA | Tb927_09_v4 | 2271690 | 2271823 | 1 | Tb09_SLRNA_0023 |  |
| SL RNA | misc RNA | Tb927_09_v4 | 2273073 | 2273206 | 1 | Tb09_SLRNA_0024 |  |
| SL RNA | misc RNA | Tb927_09_v4 | 2274435 | 2274568 | 1 | Tb09_SLRNA_0025 |  |
| SL RNA | misc RNA | Tb927_09_v4 | 2275831 | 2275964 | 1 | Tb09_SLRNA_0026 |  |
| SL RNA | misc RNA | Tb927_09_v4 | 2267562 | 2267695 | 1 | Tb09_SLRNA_0020 |  |
| SL RNA | misc RNA | Tb927_09_v4 | 2268926 | 2269059 | 1 | Tb09_SLRNA_0021 |  |
| SL RNA | misc RNA | Tb927_09_v4 | 2270283 | 2270416 | 1 | Tb09_SLRNA_0022 |  |
| SL RNA | misc RNA | Tb927_09_v4 | 2278597 | 2278651 | 1 | Tb09_SLRNA_0028 |  |
| SL RNA | misc RNA | Tb927_09_v4 | 2277213 | 2277346 | 1 | Tb09_SLRNA_0027 |  |
| tRNA Leucine | tRNA | Tb927_10_v4 | 1907650 | 1907731 | 1 | Tb10_tRNA_Leu_1 |  |
| tRNA Proline | tRNA | Tb927_04_v4 | 317914 | 317985 | -1 | Tb927.4.1195 |  |
| tRNA Glycine | tRNA | Tb927_10_v4 | 1867515 | 1867585 | 1 | Tb10_tRNA_Gly_1 |  |
| tRNA Serine | tRNA | Tb927_07_v4 | 1890653 | 1890733 | -1 | Tb927.7.6822 |  |
| tRNA Alanine | tRNA | Tb927_07_v4 | 1890511 | 1890583 | -1 | Tb927.7.6821 |  |
| tRNA Alanine | tRNA | Tb927_07_v4 | 1894352 | 1894424 | 1 | Tb927.7.6826 |  |
| tRNA Serine | tRNA | Tb927_07_v4 | 1894202 | 1894282 | 1 | Tb927.7.6825 |  |
| tRNA Aspartic acid | tRNA | Tb927_07_v4 | 1894054 | 1894125 | -1 | Tb927.7.6824 |  |
| tRNA Aspartic acid | tRNA | Tb927_07_v4 | 1890810 | 1890881 | 1 | Tb927.7.6823 |  |
| tRNA Lysine | tRNA | Tb927_07_v4 | 1910941 | 1911013 | 1 | Tb927.7.6845 |  |
| tRNA Cysteine | tRNA | Tb927_08_v4 | 1053540 | 1053611 | 1 | Tb927.8.3526 |  |
| tRNA Cysteine | tRNA | Tb927_08_v4 | 1053326 | 1053397 | -1 | Tb927.8.3523 |  |
| tRNA Glutamine | tRNA | Tb927_08_v4 | 1892449 | 1892520 | 1 | Tb927.8.6568 |  |
| tRNA Glutamine | tRNA | Tb927_08_v4 | 1892288 | 1892359 | 1 | Tb927.8.6566 |  |
| tRNA Leucine | tRNA | Tb927_08_v4 | 1892111 | 1892192 | 1 | Tb927.8.6564 |  |
| tRNA Isoleucine | tRNA | Tb927_08_v4 | 1891988 | 1892061 | 1 | Tb927.8.6562 |  |
| tRNA Tyrosine | tRNA | Tb927_04_v4 | 326098 | 326181 | -1 | Tb927.4.1219 |  |
| tRNA Histidine | tRNA | Tb927_11_01_v4 | 3452094 | 3452164 | 1 | Tb11_tRNA_His_1 |  |
| tRNA Isoleucine | tRNA | Tb927_08_v4 | 1892600 | 1892673 | 1 | Tb927.8.6572 |  |
| tRNA Threonine | tRNA | Tb927_04_v4 | 325958 | 326029 | 1 | Tb927.4.1216 |  |
| tRNA Glycine | tRNA | Tb927_09_v4 | 2036843 | 2036913 | 1 | Tb09_tRNA_Gly_1 |  |
| tRNA Asparagine | tRNA | Tb927_10_v4 | 2603416 | 2603488 | 1 | Tb10_tRNA_Asn_1 |  |
| tRNA Alanine | tRNA | Tb927_07_v4 | 2059249 | 2059320 | -1 | Tb927.7.7175 |  |
| tRNA Alanine | tRNA | Tb927_11_01_v4 | 3469847 | 3469918 | -1 | Tb11_tRNA_Ala_1 |  |
| tRNA Glutamic acid | tRNA | Tb927_05_v4 | 874266 | 874337 | 1 | Tb927.5.2765 |  |
| tRNA Arginine | tRNA | Tb927_08_v4 | 1587504 | 1587575 | 1 | Tb927.8.5345 |  |
| tRNA Arginine | tRNA | Tb927_10_v4 | 2603741 | 2603813 | 1 | Tb10_tRNA_Arg_1 |  |
| tRNA Arginine | tRNA | Tb927_10_v4 | 2604030 | 2604102 | 1 | Tb10_tRNA_Arg_2 |  |
| tRNA Glycine | tRNA | Tb927_11_01_v4 | 1874612 | 1874682 | 1 | Tb11_tRNA_Gly_1 |  |
| tRNA Glutamic acid | tRNA | Tb927_04_v4 | 860615 | 860686 | -1 | Tb927.4.3292 |  |
| tRNA Methionine | tRNA | Tb927_04_v4 | 860774 | 860846 | 1 | Tb927.4.3294 |  |
| tRNA Serine | tRNA | Tb927_04_v4 | 860890 | 860970 | -1 | Tb927.4.3296 |  |
| tRNA Valine | tRNA | Tb927_04_v4 | 861087 | 861160 | 1 | Tb927.4.3298 |  |
| tRNA Leucine | tRNA | Tb927_04_v4 | 842789 | 842868 | -1 | Tb927.4.3196 |  |
| tRNA Valine | tRNA | Tb927_04_v4 | 842652 | 842725 | -1 | Tb927.4.3193 |  |
| tRNA Methionine | tRNA | Tb927_04_v4 | 842927 | 842999 | -1 | Tb927.4.3199 |  |
| tRNA Leucine | tRNA | Tb927_03_v4 | 1233810 | 1233891 | -1 | Tb927.3.4396 |  |
| tRNA Serine | tRNA | Tb927_03_v4 | 1233524 | 1233604 | 1 | Tb927.3.4393 |  |
| tRNA Serine | tRNA | Tb927_03_v4 | 1233951 | 1234031 | -1 | Tb927.3.4399 |  |
| tRNA Glycine | tRNA | Tb927_07_v4 | 300549 | 300619 | 1 | Tb927.7.1175 |  |
| tRNA Proline | tRNA | Tb927_10_v4 | 1904001 | 1904072 | 1 | Tb10_tRNA_Pro_2 |  |
| tRNA Proline | tRNA | Tb927_10_v4 | 1907937 | 1908008 | 1 | Tb10_tRNA_Pro_1 |  |
| tRNA Glutamic acid | tRNA | Tb927_11_01_v4 | 3452226 | 3452297 | 1 | Tb11_tRNA_Glu_1 |  |
| tRNA Selenocysteine | tRNA | Tb927_04_v4 | 1287302 | 1287374 | -1 | Tb927.4.4663 | Incorrect annotation-really sRNA-76 |
| tRNA Valine | tRNA | Tb927_04_v4 | 1287445 | 1287517 | -1 | Tb927.4.4666 |  |
| tRNA Threonine | tRNA | Tb927_10_v4 | 1904133 | 1904204 | 1 | Tb10_tRNA_Thr_1 |  |
| tRNA Lysine | tRNA | Tb927_08_v4 | 861366 | 861438 | 1 | Tb927.8.2862 |  |
| tRNA Arginine | tRNA | Tb927_08_v4 | 861528 | 861599 | -1 | Tb927.8.2863 |  |
| tRNA Isoleucine | tRNA | Tb927_08_v4 | 854289 | 854362 | -1 | Tb927.8.2851 |  |
| tRNA Glutamine | tRNA | Tb927_08_v4 | 854457 | 854528 | 1 | Tb927.8.2852 |  |
| tRNA Glycine | tRNA | Tb927_08_v4 | 858106 | 858177 | 1 | Tb927.8.2856 |  |
| tRNA Valine | tRNA | Tb927_08_v4 | 854585 | 854657 | 1 | Tb927.8.2853 |  |
| tRNA Lysine | tRNA | Tb927_08_v4 | 854728 | 854800 | 1 | Tb927.8.2854 |  |
| tRNA Arginine | tRNA | Tb927_08_v4 | 858599 | 858670 | 1 | Tb927.8.2859 |  |
| tRNA Leucine | tRNA | Tb927_08_v4 | 858265 | 858346 | 1 | Tb927.8.2857 |  |
| tRNA Threonine | tRNA | Tb927_08_v4 | 858430 | 858501 | -1 | Tb927.8.2858 |  |
| tRNA Lysine | tRNA | Tb927_10_v4 | 2603873 | 2603944 | 1 | Tb10_tRNA_Lys_1 |  |
| tRNA Arginine | tRNA | Tb927_05_v4 | 138373 | 138444 | -1 | Tb927.5.445 |  |
| tRNA Methionine | tRNA | Tb927_10_v4 | 1907793 | 1907864 | -1 | Tb10_tRNA_Met_1 |  |
| tRNA Phenylalanine | tRNA | Tb927_11_01_v4 | 3452357 | 3452429 | 1 | Tb11_tRNA_Phe_1 |  |
| tRNA Phenylalanine | tRNA | Tb927_11_01_v4 | 3470168 | 3470240 | 1 | Tb11_tRNA_Phe_2 |  |
| tRNA Tryptophan | tRNA | Tb927_08_v4 | 793613 | 793684 | -1 | Tb927.8.2666 |  |
| tRNA Asparagine | tRNA | Tb927_08_v4 | 793455 | 793527 | 1 | Tb927.8.2663 |  |
| tRNA Arginine | tRNA | Tb927_11_01_v4 | 3469985 | 3470056 | -1 | Tb11_tRNA_Arg_1 |  |
| tRNA Alanine | tRNA | Tb927_10_v4 | 2603567 | 2603638 | -1 | Tb10_tRNA_Ala_1 |  |
| M4/140 small | rRNA | Tb927_01_v4 | 802312 | 802447 | 1 | tmp.1.90 |  |
| M6/70 small | rRNA | Tb927_01_v4 | 801915 | 801992 | 1 | tmp.1.80 |  |
| M2/180 small | rRNA | Tb927_01_v4 | 801414 | 801595 | 1 | tmp.1.70 |  |
| large subunit (28S beta) | rRNA | Tb927_01_v4 | 799792 | 801327 | 1 | tmp.1.60 |  |
| large subunit alpha | rRNA | Tb927_09_v4 | 2809141 | 2810459 | -1 | Tb09_rRNA_3 |  |
| large subunit gamma (M1) | rRNA | Tb927_09_v4 | 2808734 | 2808948 | -1 | Tb09_rRNA_2 |  |
| large subunit beta | rRNA | Tb927_09_v4 | 2807678 | 2808657 | -1 | Tb09_rRNA_1 |  |
| M4 | rRNA | Tb927_09_v4 | 2941359 | 2941494 | 1 | Tb09_rRNA_6 |  |
| M2 | rRNA | Tb927_09_v4 | 2940449 | 2940631 | 1 | Tb09_rRNA_4 |  |
| M6 | rRNA | Tb927_09_v4 | 2940967 | 2941044 | 1 | Tb09_rRNA_5 |  |
| large subunit epsilon (M4) | rRNA | Tb927_07_v4 | 1941008 | 1941143 | 1 | Tb927.7.6869 |  |
| large subunit delta (M2) | rRNA | Tb927_07_v4 | 1940108 | 1940290 | 1 | Tb927.7.6863 |  |
| large subunit zeta (M6) | rRNA | Tb927_07_v4 | 1940612 | 1940689 | 1 | Tb927.7.6866 |  |
| small subunit | rRNA | Tb927_07_v4 | 1951181 | 1953431 | 1 | Tb927.7.6881 |  |
| 5.8S (M3) | rRNA | Tb927_07_v4 | 1953772 | 1953943 | 1 | Tb927.7.6882 |  |
| large subunit alpha | rRNA | Tb927_07_v4 | 1954526 | 1956353 | 1 | Tb927.7.6883 |  |
| large subunit gamma (M1) | rRNA | Tb927_07_v4 | 1956546 | 1956760 | 1 | Tb927.7.6884 |  |
| large subunit beta | rRNA | Tb927_07_v4 | 1956837 | 1958321 | 1 | Tb927.7.6885 |  |
| large subunit delta (M2) | rRNA | Tb927_07_v4 | 1958459 | 1958641 | 1 | Tb927.7.6886 |  |
| large subunit zeta (M6) | rRNA | Tb927_07_v4 | 1958963 | 1959040 | 1 | Tb927.7.6887 |  |
| large subunit epsilon (M4) | rRNA | Tb927_07_v4 | 1959361 | 1959496 | 1 | Tb927.7.6888 |  |
| large subunit zeta (M4) | rRNA | Tb927_08_v4 | 122247 | 122382 | -1 | Tb927.8.443 |  |
| M6 | rRNA | Tb927_02_v4 | 260682 | 260759 | -1 | Tb927.2.1398 |  |
| large subunit delta (M2) | rRNA | Tb927_08_v4 | 123108 | 123290 | -1 | Tb927.8.449 |  |
| M4 | rRNA | Tb927_02_v4 | 260224 | 260359 | -1 | Tb927.2.1389 |  |
| large subunit zeta (M6) | rRNA | Tb927_08_v4 | 122700 | 122777 | -1 | Tb927.8.446 |  |
| 5.8S | rRNA | Tb927_01_v4 | 796692 | 797078 | 1 | tmp.1.20 |  |
| M3 | rRNA | Tb927_01_v4 | 796728 | 796899 | 1 | tmp.1.30 |  |
| large subunit (28S alpha) | rRNA | Tb927_01_v4 | 797482 | 799344 | 1 | tmp.1.40 |  |
| M1/220 small rRNA/small 1 | rRNA | Tb927_01_v4 | 799502 | 799716 | 1 | tmp.1.50 |  |
| small subunit (18S) | rRNA | Tb927_01_v4 | 794167 | 796354 | 1 | tmp.1.10 |  |
| M4 | rRNA | Tb927_02_v4 | 278563 | 278698 | -1 | Tb927.2.1480 |  |
| M6 | rRNA | Tb927_02_v4 | 279021 | 279098 | -1 | Tb927.2.1490 |  |
| 5.8S(M3) | rRNA | Tb927_02_v4 | 265783 | 265954 | -1 | Tb927.2.1443 |  |
| 18S | rRNA | Tb927_02_v4 | 266299 | 268549 | -1 | Tb927.2.1452 |  |
| 24S_B_3' | rRNA | Tb927_02_v4 | 261403 | 262886 | -1 | Tb927.2.1416 |  |
| M1 | rRNA | Tb927_02_v4 | 262963 | 263177 | -1 | Tb927.2.1425 |  |
| 24S_alpha | rRNA | Tb927_02_v4 | 263372 | 265201 | -1 | Tb927.2.1434 |  |
| M2 | rRNA | Tb927_02_v4 | 261083 | 261265 | -1 | Tb927.2.1407 |  |
| 5.8S(M3) | rRNA | Tb927_02_v4 | 284122 | 284293 | -1 | Tb927.2.1540 |  |
| rRNA | rRNA | Tb927_02_v4 | 284636 | 286886 | -1 | Tb927.2.1550 |  |
| M2 | rRNA | Tb927_02_v4 | 279422 | 279604 | -1 | Tb927.2.1500 |  |
| 24S_B_3' | rRNA | Tb927_02_v4 | 279742 | 281225 | -1 | Tb927.2.1510 |  |
| M1 | rRNA | Tb927_02_v4 | 281302 | 281516 | -1 | Tb927.2.1520 |  |
| 24S_alpha | rRNA | Tb927_02_v4 | 281711 | 283540 | -1 | Tb927.2.1530 |  |
| 5.8S | rRNA | Tb927_10_v4 | 3914467 | 3914635 | 1 | Tb10_rRNA_1 |  |
| large subunit alpha | rRNA | Tb927_10_v4 | 3915230 | 3917057 | 1 | Tb10_rRNA_2 |  |
| large subunit alpha | rRNA | Tb927_11_01_v4 | 4500868 | 4502375 | 1 | Tb11_rRNA_2 |  |
| 5.8S | rRNA | Tb927_11_01_v4 | 4500105 | 4500273 | 1 | Tb11_rRNA_1 |  |
| 5S(M5) | rRNA | Tb927_08_v4 | 458526 | 458644 | -1 | Tb927.8.1387 |  |
| 5S(M5) | rRNA | Tb927_08_v4 | 457790 | 457908 | -1 | Tb927.8.1386 |  |
| 5S(M5) | rRNA | Tb927_08_v4 | 460411 | 460441 | -1 | Tb927.8.1389 |  |
| 5S(M5) | rRNA | Tb927_08_v4 | 459261 | 459379 | -1 | Tb927.8.1388 |  |

| 5S(M5) | rRNA | Tb927_08_v4 | 455582 | 455700 | -1 | Tb927.8.1383 |  |
| --- | --- | --- | --- | --- | --- | --- | --- |
| 5S(M5) | rRNA | Tb927_08_v4 | 454846 | 454964 | -1 | Tb927.8.1382 |  |
| 5S(M5) | rRNA | Tb927_08_v4 | 457054 | 457172 | -1 | Tb927.8.1385 |  |
| 5S(M5) | rRNA | Tb927_08_v4 | 456318 | 456436 | -1 | Tb927.8.1384 |  |
| 5S(M5) | rRNA | Tb927_08_v4 | 454111 | 454229 | -1 | Tb927.8.1381 |  |
| cluster | rRNA | Tb927_01_v4 | 794148 | 802447 | 1 | tmp.1.100 |  |
| M6 | rRNA | Tb927_02_v4 | 377747 | 377824 | 1 | Tb927.2.1997 |  |
| M2 | rRNA | Tb927_02_v4 | 377241 | 377423 | 1 | Tb927.2.1986 |  |
| large subunit epsilon (M4) | rRNA | Tb927_06_v4 | 49720 | 49855 | -1 | Tb927.6.181 |  |
| large subunit zeta (M6) | rRNA | Tb927_06_v4 | 50167 | 50244 | -1 | Tb927.6.182 |  |
| large subunit delta (M2) | rRNA | Tb927_06_v4 | 50568 | 50750 | -1 | Tb927.6.183 |  |
| large subunit beta | rRNA | Tb927_06_v4 | 50888 | 51403 | -1 | Tb927.6.184 |  |
| large subunit beta | rRNA | Tb927_06_v4 | 51911 | 52891 | -1 | Tb927.6.185 |  |
| large subunit gamma (M1) | rRNA | Tb927_06_v4 | 52968 | 53182 | -1 | Tb927.6.186 |  |
| large subunit alpha | rRNA | Tb927_06_v4 | 53375 | 53706 | -1 | Tb927.6.187 |  |
| 24S_B_3' | rRNA | Tb927_02_v4 | 375619 | 377103 | 1 | Tb927.2.1975 |  |
| 18S | rRNA | Tb927_02_v4 | 369957 | 372207 | 1 | Tb927.2.1931 |  |
| M3 | rRNA | Tb927_02_v4 | 372546 | 372717 | 1 | Tb927.2.1942 |  |
| 24S_alpha | rRNA | Tb927_02_v4 | 373303 | 375132 | 1 | Tb927.2.1953 |  |
| M1 | rRNA | Tb927_02_v4 | 375328 | 375542 | 1 | Tb927.2.1964 |  |
| small subunit | rRNA | Tb927_03_v4 | 902861 | 905111 | 1 | Tb927.3.3421 |  |
| large subunit alpha | rRNA | Tb927_03_v4 | 906206 | 908032 | 1 | Tb927.3.3423 |  |
| 5.8S (M3) | rRNA | Tb927_03_v4 | 905452 | 905623 | 1 | Tb927.3.3422 |  |
| large subunit beta | rRNA | Tb927_03_v4 | 908519 | 910003 | 1 | Tb927.3.3425 |  |
| large subunit gamma (M1) | rRNA | Tb927_03_v4 | 908228 | 908442 | 1 | Tb927.3.3424 |  |
| large subunit zeta (M6) | rRNA | Tb927_03_v4 | 910655 | 910732 | 1 | Tb927.3.3427 |  |
| large subunit delta (M2) | rRNA | Tb927_03_v4 | 910141 | 910323 | 1 | Tb927.3.3426 |  |
| 5.8S (M3) | rRNA | Tb927_03_v4 | 942169 | 942340 | 1 | Tb927.3.3439 |  |
| large subunit delta (M2) | rRNA | Tb927_03_v4 | 946858 | 947040 | 1 | Tb927.3.3444 |  |
| large subunit zeta (M6) | rRNA | Tb927_03_v4 | 947368 | 947445 | 1 | Tb927.3.3445 |  |
| large subunit gamma (M1) | rRNA | Tb927_03_v4 | 944945 | 945159 | 1 | Tb927.3.3442 |  |
| large subunit beta | rRNA | Tb927_03_v4 | 945236 | 946720 | 1 | Tb927.3.3443 |  |
| 5.8S (M3) | rRNA | Tb927_03_v4 | 960539 | 960710 | 1 | Tb927.3.3448 |  |
| large subunit alpha | rRNA | Tb927_03_v4 | 961295 | 963123 | 1 | Tb927.3.3449 |  |
| large subunit epsilon (M4) | rRNA | Tb927_03_v4 | 947768 | 947903 | 1 | Tb927.3.3446 |  |
| small subunit | rRNA | Tb927_03_v4 | 957940 | 960190 | 1 | Tb927.3.3447 |  |
| large subunit alpha | rRNA | Tb927_03_v4 | 942923 | 944749 | 1 | Tb927.3.3441 |  |
| small subunit | rRNA | Tb927_03_v4 | 921223 | 923473 | 1 | Tb927.3.3429 |  |
| large subunit epsilon (M4) | rRNA | Tb927_03_v4 | 911053 | 911188 | 1 | Tb927.3.3428 |  |
| 5.8S (M3) | rRNA | Tb927_03_v4 | 923814 | 923985 | 1 | Tb927.3.3431 |  |
| large subunit alpha | rRNA | Tb927_03_v4 | 924568 | 926394 | 1 | Tb927.3.3432 |  |
| large subunit gamma (M1) | rRNA | Tb927_03_v4 | 926590 | 926804 | 1 | Tb927.3.3433 |  |
| large subunit beta | rRNA | Tb927_03_v4 | 926881 | 928365 | 1 | Tb927.3.3434 |  |
| large subunit delta (M2) | rRNA | Tb927_03_v4 | 928503 | 928685 | 1 | Tb927.3.3435 |  |
| large subunit zeta (M6) | rRNA | Tb927_03_v4 | 929008 | 929085 | 1 | Tb927.3.3436 |  |
| large subunit epsilon (M4) | rRNA | Tb927_03_v4 | 929408 | 929543 | 1 | Tb927.3.3437 |  |
| small subunit | rRNA | Tb927_03_v4 | 939578 | 941828 | 1 | Tb927.3.3438 |  |
| large subunit delta (M2) | rRNA | Tb927_03_v4 | 965232 | 965414 | 1 | Tb927.3.3453 |  |
| large subunit zeta (M6) | rRNA | Tb927_03_v4 | 965735 | 965812 | 1 | Tb927.3.3454 |  |
| large subunit epsilon (M4) | rRNA | Tb927_03_v4 | 966132 | 966267 | 1 | Tb927.3.3455 |  |
| large subunit gamma (M1) | rRNA | Tb927_03_v4 | 963319 | 963533 | 1 | Tb927.3.3451 |  |
| large subunit beta | rRNA | Tb927_03_v4 | 963610 | 965094 | 1 | Tb927.3.3452 |  |
| M4 | rRNA | Tb927_02_v4 | 378146 | 378281 | 1 | Tb927.2.2008 |  |
| TB10Cs1C1 | C/D snoRNA | Tb927_10_v4 | 1729248 | 1729342 | 1 | Tb10_snoRNA_0003 |  |
| TB10Cs1C1 | C/D snoRNA | Tb927_10_v4 | 1738983 | 1739077 | 1 | Tb10_snoRNA_0013 |  |
| TB10Cs1C1 | C/D snoRNA | Tb927_10_v4 | 1730711 | 1730805 | 1 | Tb10_snoRNA_0009 |  |
| TB10Cs1C3 | C/D snoRNA | Tb927_10_v4 | 1739913 | 1740010 | 1 | Tb10_snoRNA_0015 |  |
| TB10Cs1C3 | C/D snoRNA | Tb927_10_v4 | 1730178 | 1730275 | 1 | Tb10_snoRNA_0005 |  |
| TB10Cs1C4 | C/D snoRNA | Tb927_10_v4 | 1740226 | 1740302 | 1 | Tb10_snoRNA_0017 |  |
| TB10Cs1C4 | C/D snoRNA | Tb927_10_v4 | 1729028 | 1729104 | 1 | Tb10_snoRNA_0001 |  |
| TB10Cs1C4 | C/D snoRNA | Tb927_10_v4 | 1738763 | 1738839 | 1 | Tb10_snoRNA_0011 |  |
| TB10Cs1C4 | C/D snoRNA | Tb927_10_v4 | 1730491 | 1730567 | 1 | Tb10_snoRNA_0007 |  |
| TB10Cs1H1 | H/ACA snoRNA | Tb927_10_v4 | 1739124 | 1739196 | 1 | Tb10_snoRNA_0014 |  |
| TB10Cs1H1 | H/ACA snoRNA | Tb927_10_v4 | 1730852 | 1730924 | 1 | Tb10_snoRNA_0010 |  |
| TB10Cs1H1 | H/ACA snoRNA | Tb927_10_v4 | 1729389 | 1729461 | 1 | Tb10_snoRNA_0004 |  |
| TB10Cs1H2 | H/ACA snoRNA | Tb927_10_v4 | 1740117 | 1740183 | 1 | Tb10_snoRNA_0016 |  |
| TB10Cs1H2 | H/ACA snoRNA | Tb927_10_v4 | 1730382 | 1730448 | 1 | Tb10_snoRNA_0006 |  |
| TB10Cs1H3 | H/ACA snoRNA | Tb927_10_v4 | 1729160 | 1729228 | 1 | Tb10_snoRNA_0002 |  |
| TB10Cs1H3 | H/ACA snoRNA | Tb927_10_v4 | 1738895 | 1738963 | 1 | Tb10_snoRNA_0012 |  |
| TB10Cs1H3 | H/ACA snoRNA | Tb927_10_v4 | 1730623 | 1730691 | 1 | Tb10_snoRNA_0008 |  |
| TB10Cs2C1 | C/D snoRNA | Tb927_10_v4 | 2714935 | 2715030 | 1 | Tb10_snoRNA_0019 |  |
| TB10Cs2C1 | C/D snoRNA | Tb927_10_v4 | 2715885 | 2715980 | 1 | Tb10_snoRNA_0025 |  |
| TB10Cs2C1 | C/D snoRNA | Tb927_10_v4 | 2715410 | 2715505 | 1 | Tb10_snoRNA_0022 |  |
| TB10Cs2C2 | C/D snoRNA | Tb927_10_v4 | 2716147 | 2716216 | 1 | Tb10_snoRNA_0027 |  |
| TB10Cs2C2 | C/D snoRNA | Tb927_10_v4 | 2715672 | 2715741 | 1 | Tb10_snoRNA_0024 |  |
| TB10Cs2C2 | C/D snoRNA | Tb927_10_v4 | 2715197 | 2715266 | 1 | Tb10_snoRNA_0021 |  |
| TB10Cs2H1 | H/ACA snoRNA | Tb927_10_v4 | 2716265 | 2716323 | 1 | Tb10_snoRNA_0028 |  |
| TB10Cs2H1 | H/ACA snoRNA | Tb927_10_v4 | 2714834 | 2714901 | 1 | Tb10_snoRNA_0018 |  |
| TB10Cs2H2 | H/ACA snoRNA | Tb927_10_v4 | 2716064 | 2716129 | 1 | Tb10_snoRNA_0026 |  |
| TB10Cs2H2 | H/ACA snoRNA | Tb927_10_v4 | 2715589 | 2715654 | 1 | Tb10_snoRNA_0023 |  |
| TB10Cs2H2 | H/ACA snoRNA | Tb927_10_v4 | 2715114 | 2715179 | 1 | Tb10_snoRNA_0020 |  |
| TB10Cs3C1 | C/D snoRNA | Tb927_10_v4 | 3814615 | 3814725 | 1 | Tb10_snoRNA_0081 |  |
| TB10Cs3C1 | C/D snoRNA | Tb927_10_v4 | 3813407 | 3813517 | 1 | Tb10_snoRNA_0074 |  |
| TB10Cs3C1 | C/D snoRNA | Tb927_10_v4 | 3812199 | 3812309 | 1 | Tb10_snoRNA_0067 |  |
| TB10Cs3C1 | C/D snoRNA | Tb927_10_v4 | 3810991 | 3811101 | 1 | Tb10_snoRNA_0060 |  |
| TB10Cs3C2 | C/D snoRNA | Tb927_10_v4 | 3814820 | 3814920 | 1 | Tb10_snoRNA_0083 |  |
| TB10Cs3C2 | C/D snoRNA | Tb927_10_v4 | 3813612 | 3813712 | 1 | Tb10_snoRNA_0076 |  |
| TB10Cs3C2 | C/D snoRNA | Tb927_10_v4 | 3812404 | 3812504 | 1 | Tb10_snoRNA_0069 |  |
| TB10Cs3C2 | C/D snoRNA | Tb927_10_v4 | 3811196 | 3811296 | 1 | Tb10_snoRNA_0062 |  |
| TB10Cs3C3 | C/D snoRNA | Tb927_10_v4 | 3810355 | 3810459 | 1 | Tb10_snoRNA_0056 |  |
| TB10Cs3C3 | C/D snoRNA | Tb927_10_v4 | 3815187 | 3815235 | 1 | Tb10_snoRNA_0084 |  |
| TB10Cs3C3 | C/D snoRNA | Tb927_10_v4 | 3813979 | 3814083 | 1 | Tb10_snoRNA_0077 |  |
| TB10Cs3C3 | C/D snoRNA | Tb927_10_v4 | 3812771 | 3812875 | 1 | Tb10_snoRNA_0070 |  |
| TB10Cs3C3 | C/D snoRNA | Tb927_10_v4 | 3811563 | 3811667 | 1 | Tb10_snoRNA_0063 |  |
| TB10Cs3C4 | C/D snoRNA | Tb927_10_v4 | 3810497 | 3810578 | 1 | Tb10_snoRNA_0057 |  |
| TB10Cs3C4 | C/D snoRNA | Tb927_10_v4 | 3814121 | 3814202 | 1 | Tb10_snoRNA_0078 |  |
| TB10Cs3C4 | C/D snoRNA | Tb927_10_v4 | 3812913 | 3812994 | 1 | Tb10_snoRNA_0071 |  |
| TB10Cs3C4 | C/D snoRNA | Tb927_10_v4 | 3811705 | 3811786 | 1 | Tb10_snoRNA_0064 |  |
| TB10Cs3C5 | C/D snoRNA | Tb927_10_v4 | 3810773 | 3810871 | 1 | Tb10_snoRNA_0058 |  |
| TB10Cs3C5 | C/D snoRNA | Tb927_10_v4 | 3814397 | 3814495 | 1 | Tb10_snoRNA_0079 |  |
| TB10Cs3C5 | C/D snoRNA | Tb927_10_v4 | 3813189 | 3813287 | 1 | Tb10_snoRNA_0072 |  |
| TB10Cs3C5 | C/D snoRNA | Tb927_10_v4 | 3811981 | 3812079 | 1 | Tb10_snoRNA_0065 |  |
| TB10Cs3H1 | H/ACA snoRNA | Tb927_10_v4 | 3814741 | 3814804 | 1 | Tb10_snoRNA_0082 |  |
| TB10Cs3H1 | H/ACA snoRNA | Tb927_10_v4 | 3813533 | 3813596 | 1 | Tb10_snoRNA_0075 |  |
| TB10Cs3H1 | H/ACA snoRNA | Tb927_10_v4 | 3812325 | 3812388 | 1 | Tb10_snoRNA_0068 |  |
| TB10Cs3H1 | H/ACA snoRNA | Tb927_10_v4 | 3811117 | 3811180 | 1 | Tb10_snoRNA_0061 |  |
| TB10Cs3H2 | H/ACA snoRNA | Tb927_10_v4 | 3814533 | 3814598 | 1 | Tb10_snoRNA_0080 |  |
| TB10Cs3H2 | H/ACA snoRNA | Tb927_10_v4 | 3813325 | 3813390 | 1 | Tb10_snoRNA_0073 |  |
| TB10Cs3H2 | H/ACA snoRNA | Tb927_10_v4 | 3810909 | 3810974 | 1 | Tb10_snoRNA_0059 |  |
| TB10Cs3H2 | H/ACA snoRNA | Tb927_10_v4 | 3812117 | 3812182 | 1 | Tb10_snoRNA_0066 |  |
| TB10Cs4C1 | C/D snoRNA | Tb927_10_v4 | 3204052 | 3204129 | -1 | Tb10_snoRNA_0043 |  |
| TB10Cs4C2 | C/D snoRNA | Tb927_10_v4 | 3273960 | 3274046 | -1 | Tb10_snoRNA_0048 |  |
| TB10Cs4C2 | C/D snoRNA | Tb927_10_v4 | 3327778 | 3327843 | -1 | Tb10_snoRNA_0055 |  |
| TB10Cs4C2 | C/D snoRNA | Tb927_10_v4 | 3203632 | 3203718 | -1 | Tb10_snoRNA_0040 |  |
| TB10Cs4C2 | C/D snoRNA | Tb927_10_v4 | 3197724 | 3197810 | -1 | Tb10_snoRNA_0033 |  |
| TB10Cs4C3 | C/D snoRNA | Tb927_10_v4 | 3327602 | 3327681 | -1 | Tb10_snoRNA_0054 |  |
| TB10Cs4C3 | C/D snoRNA | Tb927_10_v4 | 3203456 | 3203535 | -1 | Tb10_snoRNA_0039 |  |
| TB10Cs4C3 | C/D snoRNA | Tb927_10_v4 | 3273784 | 3273863 | -1 | Tb10_snoRNA_0047 |  |
| TB10Cs4C3 | C/D snoRNA | Tb927_10_v4 | 3197548 | 3197627 | -1 | Tb10_snoRNA_0032 |  |
| TB10Cs4C4 | C/D snoRNA | Tb927_10_v4 | 3327336 | 3327431 | -1 | Tb10_snoRNA_0052 |  |
| TB10Cs4C4 | C/D snoRNA | Tb927_10_v4 | 3203190 | 3203285 | -1 | Tb10_snoRNA_0037 |  |
| TB10Cs4C4 | C/D snoRNA | Tb927_10_v4 | 3273518 | 3273613 | -1 | Tb10_snoRNA_0045 |  |
| TB10Cs4C4 | C/D snoRNA | Tb927_10_v4 | 3197282 | 3197377 | -1 | Tb10_snoRNA_0030 |  |
| TB10Cs4C5 | C/D snoRNA | Tb927_10_v4 | 3326970 | 3327055 | -1 | Tb10_snoRNA_0050 |  |
| TB10Cs4C5 | C/D snoRNA | Tb927_10_v4 | 3202824 | 3202909 | -1 | Tb10_snoRNA_0035 |  |
| TB10Cs4H1 | H/ACA snoRNA | Tb927_10_v4 | 3203940 | 3204016 | -1 | Tb10_snoRNA_0042 |  |
| TB10Cs4H2 | H/ACA snoRNA | Tb927_10_v4 | 3274063 | 3274127 | -1 | Tb10_snoRNA_0049 |  |
| TB10Cs4H2 | H/ACA snoRNA | Tb927_10_v4 | 3203735 | 3203803 | -1 | Tb10_snoRNA_0041 |  |
| TB10Cs4H2 | H/ACA snoRNA | Tb927_10_v4 | 3197827 | 3197891 | -1 | Tb10_snoRNA_0034 |  |
| TB10Cs4H3 | H/ACA snoRNA | Tb927_10_v4 | 3327446 | 3327514 | -1 | Tb10_snoRNA_0053 |  |
| TB10Cs4H3 | H/ACA snoRNA | Tb927_10_v4 | 3203300 | 3203368 | -1 | Tb10_snoRNA_0038 |  |
| TB10Cs4H3 | H/ACA snoRNA | Tb927_10_v4 | 3273628 | 3273696 | -1 | Tb10_snoRNA_0046 |  |
| TB10Cs4H3 | H/ACA snoRNA | Tb927_10_v4 | 3197392 | 3197460 | -1 | Tb10_snoRNA_0031 |  |
| TB10Cs4H4 | H/ACA snoRNA | Tb927_10_v4 | 3327216 | 3327282 | -1 | Tb10_snoRNA_0051 |  |
| TB10Cs4H4 | H/ACA snoRNA | Tb927_10_v4 | 3273398 | 3273464 | -1 | Tb10_snoRNA_0044 |  |
| TB10Cs4H4 | H/ACA snoRNA | Tb927_10_v4 | 3197162 | 3197228 | -1 | Tb10_snoRNA_0029 |  |
| TB10Cs4H4 | H/ACA snoRNA | Tb927_10_v4 | 3203070 | 3203136 | -1 | Tb10_snoRNA_0036 |  |
| TB11Cs1C1 | C/D snoRNA | Tb927_11_01_v4 | 2142084 | 2142159 | -1 | Tb11_snoRNA_0037 |  |
| TB11Cs1C2 | C/D snoRNA | Tb927_11_01_v4 | 2141548 | 2141633 | -1 | Tb11_snoRNA_0036 |  |
| TB11Cs1C2 | C/D snoRNA | Tb927_11_01_v4 | 2134730 | 2134815 | -1 | Tb11_snoRNA_0034 |  |
| TB11Cs1C3 | C/D snoRNA | Tb927_11_01_v4 | 2141370 | 2141447 | -1 | Tb11_snoRNA_0035 |  |
| TB11Cs1C3 | C/D snoRNA | Tb927_11_01_v4 | 2134558 | 2134635 | -1 | Tb11_snoRNA_0033 |  |
| TB11Cs2C1 | C/D snoRNA | Tb927_11_01_v4 | 2702863 | 2702937 | 1 | Tb11_snoRNA_0039 |  |
| TB11Cs2C1 | C/D snoRNA | Tb927_11_01_v4 | 2703685 | 2703759 | 1 | Tb11_snoRNA_0042 |  |
| TB11Cs2C2 | C/D snoRNA | Tb927_11_01_v4 | 2703912 | 2704001 | 1 | Tb11_snoRNA_0043 |  |
| TB11Cs2C2 | C/D snoRNA | Tb927_11_01_v4 | 2703080 | 2703169 | 1 | Tb11_snoRNA_0040 |  |
| TB11Cs2H1 | H/ACA snoRNA | Tb927_11_01_v4 | 2702551 | 2702733 | 1 | Tb11_snoRNA_0038 |  |
| TB11Cs2H1 | H/ACA snoRNA | Tb927_11_01_v4 | 2703287 | 2703555 | 1 | Tb11_snoRNA_0041 |  |
| TB11Cs3C1 | C/D snoRNA | Tb927_11_01_v4 | 2706383 | 2706474 | 1 | Tb11_snoRNA_0045 |  |
| TB11Cs3C2 | C/D snoRNA | Tb927_11_01_v4 | 2706539 | 2706641 | 1 | Tb11_snoRNA_0046 |  |
| TB11Cs3H1 | H/ACA snoRNA | Tb927_11_01_v4 | 2706239 | 2706310 | 1 | Tb11_snoRNA_0044 |  |
| TB11Cs3H2 | H/ACA snoRNA | Tb927_11_01_v4 | 2707090 | 2707164 | 1 | Tb11_snoRNA_0047 |  |
| TB11Cs4C1 | C/D snoRNA | Tb927_11_01_v4 | 457199 | 457288 | -1 | Tb11_snoRNA_0005 |  |
| TB11Cs4C1 | C/D snoRNA | Tb927_11_01_v4 | 458183 | 458272 | -1 | Tb11_snoRNA_0011 |  |
| TB11Cs4C1 | C/D snoRNA | Tb927_11_01_v4 | 459170 | 459259 | -1 | Tb11_snoRNA_0017 |  |
| TB11Cs4C1 | C/D snoRNA | Tb927_11_01_v4 | 460159 | 460248 | -1 | Tb11_snoRNA_0023 |  |
| TB11Cs4C1 | C/D snoRNA | Tb927_11_01_v4 | 461148 | 461237 | -1 | Tb11_snoRNA_0029 |  |
| TB11Cs4C2 | C/D snoRNA | Tb927_11_01_v4 | 456973 | 457069 | -1 | Tb11_snoRNA_0004 |  |
| TB11Cs4C2 | C/D snoRNA | Tb927_11_01_v4 | 457963 | 458059 | -1 | Tb11_snoRNA_0010 |  |
| TB11Cs4C2 | C/D snoRNA | Tb927_11_01_v4 | 458949 | 459045 | -1 | Tb11_snoRNA_0016 |  |
| TB11Cs4C2 | C/D snoRNA | Tb927_11_01_v4 | 459933 | 460029 | -1 | Tb11_snoRNA_0022 |  |
| TB11Cs4C2 | C/D snoRNA | Tb927_11_01_v4 | 460922 | 461018 | -1 | Tb11_snoRNA_0028 |  |
| TB11Cs4C3 | C/D snoRNA | Tb927_11_01_v4 | 456621 | 456721 | -1 | Tb11_snoRNA_0002 |  |
| TB11Cs4C3 | C/D snoRNA | Tb927_11_01_v4 | 457612 | 457712 | -1 | Tb11_snoRNA_0008 |  |
| TB11Cs4C3 | C/D snoRNA | Tb927_11_01_v4 | 458596 | 458696 | -1 | Tb11_snoRNA_0014 |  |
| TB11Cs4C3 | C/D snoRNA | Tb927_11_01_v4 | 459583 | 459683 | -1 | Tb11_snoRNA_0020 |  |
| TB11Cs4C3 | C/D snoRNA | Tb927_11_01_v4 | 460572 | 460672 | -1 | Tb11_snoRNA_0026 |  |
| TB11Cs4C3 | C/D snoRNA | Tb927_11_01_v4 | 461561 | 461605 | -1 | Tb11_snoRNA_0032 |  |
| TB11Cs4H1 | H/ACA snoRNA | Tb927_11_01_v4 | 457782 | 457855 | -1 | Tb11_snoRNA_0009 |  |
| TB11Cs4H1 | H/ACA snoRNA | Tb927_11_01_v4 | 456792 | 456864 | -1 | Tb11_snoRNA_0003 |  |
| TB11Cs4H1 | H/ACA snoRNA | Tb927_11_01_v4 | 458767 | 458839 | -1 | Tb11_snoRNA_0015 |  |
| TB11Cs4H1 | H/ACA snoRNA | Tb927_11_01_v4 | 459754 | 459826 | -1 | Tb11_snoRNA_0021 |  |
| TB11Cs4H1 | H/ACA snoRNA | Tb927_11_01_v4 | 460742 | 460815 | -1 | Tb11_snoRNA_0027 |  |
| TB11Cs4H2 | H/ACA snoRNA | Tb927_11_01_v4 | 456463 | 456528 | -1 | Tb11_snoRNA_0001 |  |
| TB11Cs4H2 | H/ACA snoRNA | Tb927_11_01_v4 | 457454 | 457519 | -1 | Tb11_snoRNA_0007 |  |
| TB11Cs4H2 | H/ACA snoRNA | Tb927_11_01_v4 | 458438 | 458503 | -1 | Tb11_snoRNA_0013 |  |
| TB11Cs4H2 | H/ACA snoRNA | Tb927_11_01_v4 | 459425 | 459490 | -1 | Tb11_snoRNA_0019 |  |
| TB11Cs4H2 | H/ACA snoRNA | Tb927_11_01_v4 | 460414 | 460479 | -1 | Tb11_snoRNA_0025 |  |
| TB11Cs4H2 | H/ACA snoRNA | Tb927_11_01_v4 | 461403 | 461468 | -1 | Tb11_snoRNA_0031 |  |
| TB11Cs4H3 | H/ACA snoRNA | Tb927_11_01_v4 | 457326 | 457403 | -1 | Tb11_snoRNA_0006 |  |
| TB11Cs4H3 | H/ACA snoRNA | Tb927_11_01_v4 | 458310 | 458387 | -1 | Tb11_snoRNA_0012 |  |
| TB11Cs4H3 | H/ACA snoRNA | Tb927_11_01_v4 | 459297 | 459374 | -1 | Tb11_snoRNA_0018 |  |
| TB11Cs4H3 | H/ACA snoRNA | Tb927_11_01_v4 | 460286 | 460363 | -1 | Tb11_snoRNA_0024 |  |
| TB11Cs4H3 | H/ACA snoRNA | Tb927_11_01_v4 | 461275 | 461352 | -1 | Tb11_snoRNA_0030 |  |
| TB3Cs2C1 | C/D snoRNA | Tb927_03_v4 | 494356 | 494433 | -1 | Tb927.3.1892 |  |
| TB3Cs2C1 | C/D snoRNA | Tb927_03_v4 | 494224 | 494301 | -1 | Tb927.3.1891 |  |
| TB3Cs2C1 | C/D snoRNA | Tb927_03_v4 | 494596 | 494673 | -1 | Tb927.3.1894 |  |
| TB3Cs2C1 | C/D snoRNA | Tb927_03_v4 | 494488 | 494539 | -1 | Tb927.3.1893 |  |
| TB3Cs2C1 | C/D snoRNA | Tb927_03_v4 | 494860 | 494965 | -1 | Tb927.3.1896 |  |
| TB3Cs2C1 | C/D snoRNA | Tb927_03_v4 | 494728 | 494805 | -1 | Tb927.3.1895 |  |
| TB3Cs2C1 | C/D snoRNA | Tb927_03_v4 | 495034 | 495139 | -1 | Tb927.3.1905 |  |
| TB5Cs1C1 | C/D snoRNA | Tb927_05_v4 | 745813 | 745899 | 1 | Tb927.5.2346 |  |
| TB5Cs1C1 | C/D snoRNA | Tb927_05_v4 | 745620 | 745703 | 1 | Tb927.5.2343 |  |
| TB5Cs1C1 | C/D snoRNA | Tb927_05_v4 | 647139 | 647225 | -1 | Tb927.5.2035 |  |
| TB6C2C1 | C/D snoRNA | Tb927_06_v4 | 806685 | 806774 | 1 | Tb927.6.2683 |  |
| TB6C2C2 | C/D snoRNA | Tb927_06_v4 | 807371 | 807469 | 1 | Tb927.6.2689 |  |
| TB6Cs1C1 | C/D snoRNA | Tb927_06_v4 | 617610 | 617702 | -1 | Tb927.6.1881 |  |
| TB6Cs1C1 | C/D snoRNA | Tb927_06_v4 | 618682 | 618774 | -1 | Tb927.6.1888 |  |
| TB6Cs1C1 | C/D snoRNA | Tb927_06_v4 | 616539 | 616631 | -1 | Tb927.6.1873 |  |
| TB6Cs1C1 | C/D snoRNA | Tb927_06_v4 | 615466 | 615558 | -1 | Tb927.6.1865 |  |
| TB6Cs1C1 | C/D snoRNA | Tb927_06_v4 | 614393 | 614485 | -1 | Tb927.6.1857 |  |
| TB6Cs1C2 | C/D snoRNA | Tb927_06_v4 | 618076 | 618139 | -1 | Tb927.6.1885 |  |
| TB6Cs1C2 | C/D snoRNA | Tb927_06_v4 | 617005 | 617068 | -1 | Tb927.6.1877 |  |
| TB6Cs1C2 | C/D snoRNA | Tb927_06_v4 | 615934 | 615997 | -1 | Tb927.6.1869 |  |
| TB6Cs1C2 | C/D snoRNA | Tb927_06_v4 | 614861 | 614924 | -1 | Tb927.6.1862 |  |
| TB6Cs1C2 | C/D snoRNA | Tb927_06_v4 | 613788 | 613851 | -1 | Tb927.6.1854 |  |
| TB6Cs1C3 | C/D snoRNA | Tb927_06_v4 | 617733 | 617821 | -1 | Tb927.6.1882 |  |
| TB6Cs1C3 | C/D snoRNA | Tb927_06_v4 | 618805 | 618893 | -1 | Tb927.6.1889 |  |
| TB6Cs1C3 | C/D snoRNA | Tb927_06_v4 | 616662 | 616750 | -1 | Tb927.6.1874 |  |
| TB6Cs1C3 | C/D snoRNA | Tb927_06_v4 | 615589 | 615677 | -1 | Tb927.6.1866 |  |
| TB6Cs1C3 | C/D snoRNA | Tb927_06_v4 | 614516 | 614604 | -1 | Tb927.6.1858 |  |
| TB6Cs1C3 | C/D snoRNA | Tb927_06_v4 | 613443 | 613531 | -1 | Tb927.6.1851 |  |
| TB6Cs1H1 | H/ACA snoRNA | Tb927_06_v4 | 618468 | 618535 | -1 | Tb927.6.1887 |  |
| TB6Cs1H1 | H/ACA snoRNA | Tb927_06_v4 | 616326 | 616393 | -1 | Tb927.6.1872 |  |
| TB6Cs1H1 | C/D snoRNA | Tb927_06_v4 | 617397 | 617464 | -1 | Tb927.6.1879 |  |
| TB6Cs1H2 | H/ACA snoRNA | Tb927_06_v4 | 618296 | 618367 | -1 | Tb927.6.1886 |  |
| TB6Cs1H2 | H/ACA snoRNA | Tb927_06_v4 | 616154 | 616225 | -1 | Tb927.6.1871 |  |
| TB6Cs1H2 | C/D snoRNA | Tb927_06_v4 | 617225 | 617296 | -1 | Tb927.6.1878 |  |
| TB6Cs1H2 | H/ACA snoRNA | Tb927_06_v4 | 615081 | 615152 | -1 | Tb927.6.1863 |  |
| TB6Cs1H2 | H/ACA snoRNA | Tb927_06_v4 | 615253 | 615320 | -1 | Tb927.6.1864 |  |
| TB6Cs1H2 | H/ACA snoRNA | Tb927_06_v4 | 614180 | 614247 | -1 | Tb927.6.1856 |  |
| TB6Cs1H2 | H/ACA snoRNA | Tb927_06_v4 | 614008 | 614079 | -1 | Tb927.6.1855 |  |
| TB6Cs1H3 | H/ACA snoRNA | Tb927_06_v4 | 619052 | 619119 | -1 | Tb927.6.1892 |  |
| TB6Cs1H3 | H/ACA snoRNA | Tb927_06_v4 | 617978 | 618045 | -1 | Tb927.6.1884 |  |
| TB6Cs1H3 | H/ACA snoRNA | Tb927_06_v4 | 616907 | 616974 | -1 | Tb927.6.1876 |  |
| TB6Cs1H3 | H/ACA snoRNA | Tb927_06_v4 | 614763 | 614830 | -1 | Tb927.6.1861 |  |
| TB6Cs1H3 | H/ACA snoRNA | Tb927_06_v4 | 615836 | 615903 | -1 | Tb927.6.1868 |  |
| TB6Cs1H3 | H/ACA snoRNA | Tb927_06_v4 | 613690 | 613757 | -1 | Tb927.6.1853 |  |
| TB6Cs1H4 | H/ACA snoRNA | Tb927_06_v4 | 618961 | 619037 | -1 | Tb927.6.1891 |  |
| TB6Cs1H4 | H/ACA snoRNA | Tb927_06_v4 | 617887 | 617963 | -1 | Tb927.6.1883 |  |
| TB6Cs1H4 | H/ACA snoRNA | Tb927_06_v4 | 616816 | 616892 | -1 | Tb927.6.1875 |  |
| TB6Cs1H4 | H/ACA snoRNA | Tb927_06_v4 | 615745 | 615821 | -1 | Tb927.6.1867 |  |
| TB6Cs1H4 | H/ACA snoRNA | Tb927_06_v4 | 614672 | 614748 | -1 | Tb927.6.1859 |  |
| TB6Cs1H4 | H/ACA snoRNA | Tb927_06_v4 | 613599 | 613675 | -1 | Tb927.6.1852 |  |
| TB6Cs2H1 | H/ACA snoRNA | Tb927_06_v4 | 806841 | 806911 | 1 | Tb927.6.2686 |  |
| TB8Cs1C1 | C/D snoRNA | Tb927_08_v4 | 536230 | 536312 | 1 | Tb927.8.1619 |  |
| TB8Cs1C1 | C/D snoRNA | Tb927_08_v4 | 536879 | 536961 | 1 | Tb927.8.1624 |  |
| TB8Cs1C1 | C/D snoRNA | Tb927_08_v4 | 534283 | 534365 | 1 | Tb927.8.1606 |  |
| TB8Cs1C1 | C/D snoRNA | Tb927_08_v4 | 533634 | 533716 | 1 | Tb927.8.1602 |  |
| TB8Cs1C1 | C/D snoRNA | Tb927_08_v4 | 535581 | 535663 | 1 | Tb927.8.1615 |  |
| TB8Cs1C1 | C/D snoRNA | Tb927_08_v4 | 534932 | 535014 | 1 | Tb927.8.1611 |  |
| TB8Cs1C2 | C/D snoRNA | Tb927_08_v4 | 536405 | 536478 | 1 | Tb927.8.1621 |  |
| TB8Cs1C2 | C/D snoRNA | Tb927_08_v4 | 533809 | 533882 | 1 | Tb927.8.1603 |  |
| TB8Cs1C2 | C/D snoRNA | Tb927_08_v4 | 534458 | 534531 | 1 | Tb927.8.1607 |  |
| TB8Cs1C2 | C/D snoRNA | Tb927_08_v4 | 535756 | 535829 | 1 | Tb927.8.1616 |  |
| TB8Cs1C2 | C/D snoRNA | Tb927_08_v4 | 535107 | 535180 | 1 | Tb927.8.1612 |  |
| TB8Cs1C3 | C/D snoRNA | Tb927_08_v4 | 536514 | 536631 | 1 | Tb927.8.1622 |  |
| TB8Cs1C3 | C/D snoRNA | Tb927_08_v4 | 533918 | 534035 | 1 | Tb927.8.1604 |  |
| TB8Cs1C3 | C/D snoRNA | Tb927_08_v4 | 534567 | 534684 | 1 | Tb927.8.1608 |  |
| TB8Cs1C3 | C/D snoRNA | Tb927_08_v4 | 535865 | 535982 | 1 | Tb927.8.1617 |  |
| TB8Cs1C3 | C/D snoRNA | Tb927_08_v4 | 535216 | 535333 | 1 | Tb927.8.1613 |  |
| TB8Cs1C4 | C/D snoRNA | Tb927_08_v4 | 536125 | 536202 | 1 | Tb927.8.1618 |  |
| TB8Cs1C4 | C/D snoRNA | Tb927_08_v4 | 536774 | 536851 | 1 | Tb927.8.1623 |  |
| TB8Cs1C4 | C/D snoRNA | Tb927_08_v4 | 534178 | 534255 | 1 | Tb927.8.1605 |  |
| TB8Cs1C4 | C/D snoRNA | Tb927_08_v4 | 533529 | 533606 | 1 | Tb927.8.1601 |  |
| TB8Cs1C4 | C/D snoRNA | Tb927_08_v4 | 534827 | 534904 | 1 | Tb927.8.1609 |  |
| TB8Cs1C4 | C/D snoRNA | Tb927_08_v4 | 535476 | 535553 | 1 | Tb927.8.1614 |  |
| TB8Cs2C0 | C/D snoRNA | Tb927_08_v4 | 549674 | 549769 | 1 | Tb927.8.1651 |  |
| TB8Cs2C1 | C/D snoRNA | Tb927_08_v4 | 549933 | 550026 | 1 | Tb927.8.1653 |  |
| TB8Cs2C1 | C/D snoRNA | Tb927_08_v4 | 550311 | 550404 | 1 | Tb927.8.1655 |  |
| TB8Cs2C1 | C/D snoRNA | Tb927_08_v4 | 550689 | 550782 | 1 | Tb927.8.1657 |  |
| TB8Cs2H1 | H/ACA snoRNA | Tb927_08_v4 | 549815 | 549881 | 1 | Tb927.8.1652 |  |
| TB8Cs2H1 | H/ACA snoRNA | Tb927_08_v4 | 550193 | 550259 | 1 | Tb927.8.1654 |  |
| TB8Cs2H1 | H/ACA snoRNA | Tb927_08_v4 | 550571 | 550637 | 1 | Tb927.8.1656 |  |
| TB8Cs2H1 | H/ACA snoRNA | Tb927_08_v4 | 550949 | 551015 | 1 | Tb927.8.1658 |  |
| TB8Cs3C1 | C/D snoRNA | Tb927_08_v4 | 653501 | 653583 | 1 | Tb927.8.2062 |  |
| TB8Cs3C1 | C/D snoRNA | Tb927_08_v4 | 657195 | 657277 | 1 | Tb927.8.2083 |  |
| TB8Cs3C1 | C/D snoRNA | Tb927_08_v4 | 691295 | 691377 | 1 | Tb927.8.2252 |  |
| TB8Cs3C2 | C/D snoRNA | Tb927_08_v4 | 653618 | 653704 | 1 | Tb927.8.2064 |  |
| TB8Cs3C2 | C/D snoRNA | Tb927_08_v4 | 657312 | 657398 | 1 | Tb927.8.2086 |  |
| TB8Cs3C2 | C/D snoRNA | Tb927_08_v4 | 691412 | 691498 | 1 | Tb927.8.2254 |  |
| TB8Cs3C3 | C/D snoRNA | Tb927_08_v4 | 653762 | 653843 | 1 | Tb927.8.2066 |  |
| TB8Cs3C3 | C/D snoRNA | Tb927_08_v4 | 657456 | 657537 | 1 | Tb927.8.2089 |  |
| TB8Cs3C3 | C/D snoRNA | Tb927_08_v4 | 691556 | 691637 | 1 | Tb927.8.2256 |  |
| TB8Cs3H1 | H/ACA snoRNA | Tb927_08_v4 | 654055 | 654119 | 1 | Tb927.8.2068 |  |
| TB8Cs3H1 | H/ACA snoRNA | Tb927_08_v4 | 655717 | 655781 | 1 | Tb927.8.2075 |  |
| TB8Cs3H1 | H/ACA snoRNA | Tb927_08_v4 | 691849 | 691913 | 1 | Tb927.8.2258 |  |
| TB8Cs3H1 | H/ACA snoRNA | Tb927_08_v4 | 693512 | 693576 | 1 | Tb927.8.2265 |  |
| TB8Cs3H1 | H/ACA snoRNA | Tb927_08_v4 | 695175 | 695239 | 1 | Tb927.8.2275 |  |
| TB9Cs1C1 | C/D snoRNA | Tb927_09_v4 | 616179 | 616275 | -1 | Tb09_snoRNA_0005 |  |
| TB9Cs1C1 | C/D snoRNA | Tb927_09_v4 | 615669 | 615765 | -1 | Tb09_snoRNA_0001 |  |
| TB9Cs1H1 | H/ACA snoRNA | Tb927_09_v4 | 615965 | 616032 | -1 | Tb09_snoRNA_0003 |  |
| TB9Cs1H1 | H/ACA snoRNA | Tb927_09_v4 | 616475 | 616542 | -1 | Tb09_snoRNA_0007 |  |
| TB9Cs1H2 | H/ACA snoRNA | Tb927_09_v4 | 615779 | 615849 | -1 | Tb09_snoRNA_0002 |  |
| TB9Cs1H2 | H/ACA snoRNA | Tb927_09_v4 | 616289 | 616359 | -1 | Tb09_snoRNA_0006 |  |
| TB9Cs1H3 | H/ACA snoRNA | Tb927_09_v4 | 616043 | 616109 | -1 | Tb09_snoRNA_0004 |  |
| TB9Cs1H3 | H/ACA snoRNA | Tb927_09_v4 | 616553 | 616608 | -1 | Tb09_snoRNA_0008 |  |
| TB9Cs2C1 | C/D snoRNA | Tb927_09_v4 | 1633028 | 1633128 | -1 | Tb09_snoRNA_0064 |  |
| TB9Cs2C1 | C/D snoRNA | Tb927_09_v4 | 1634768 | 1634868 | -1 | Tb09_snoRNA_0073 |  |
| TB9Cs2C1 | C/D snoRNA | Tb927_09_v4 | 1629550 | 1629650 | -1 | Tb09_snoRNA_0046 |  |
| TB9Cs2C1 | C/D snoRNA | Tb927_09_v4 | 1631290 | 1631390 | -1 | Tb09_snoRNA_0055 |  |
| TB9Cs2C1 | C/D snoRNA | Tb927_09_v4 | 1626064 | 1626164 | -1 | Tb09_snoRNA_0028 |  |
| TB9Cs2C1 | C/D snoRNA | Tb927_09_v4 | 1627810 | 1627910 | -1 | Tb09_snoRNA_0037 |  |
| TB9Cs2C1 | C/D snoRNA | Tb927_09_v4 | 1624312 | 1624412 | -1 | Tb09_snoRNA_0019 |  |
| TB9Cs2C1 | C/D snoRNA | Tb927_09_v4 | 1622572 | 1622672 | -1 | Tb09_snoRNA_0010 |  |
| TB9Cs2C2 | C/D snoRNA | Tb927_09_v4 | 1632791 | 1632877 | -1 | Tb09_snoRNA_0063 |  |
| TB9Cs2C2 | C/D snoRNA | Tb927_09_v4 | 1634531 | 1634617 | -1 | Tb09_snoRNA_0072 |  |
| TB9Cs2C2 | C/D snoRNA | Tb927_09_v4 | 1629313 | 1629399 | -1 | Tb09_snoRNA_0045 |  |
| TB9Cs2C2 | C/D snoRNA | Tb927_09_v4 | 1631053 | 1631139 | -1 | Tb09_snoRNA_0054 |  |
| TB9Cs2C2 | C/D snoRNA | Tb927_09_v4 | 1625827 | 1625913 | -1 | Tb09_snoRNA_0027 |  |
| TB9Cs2C2 | C/D snoRNA | Tb927_09_v4 | 1627573 | 1627659 | -1 | Tb09_snoRNA_0036 |  |
| TB9Cs2C2 | C/D snoRNA | Tb927_09_v4 | 1622335 | 1622421 | -1 | Tb09_snoRNA_0009 |  |
| TB9Cs2C2 | C/D snoRNA | Tb927_09_v4 | 1624075 | 1624161 | -1 | Tb09_snoRNA_0018 |  |
| TB9Cs2C3 | C/D snoRNA | Tb927_09_v4 | 1632598 | 1632694 | -1 | Tb09_snoRNA_0062 |  |
| TB9Cs2C3 | C/D snoRNA | Tb927_09_v4 | 1634336 | 1634432 | -1 | Tb09_snoRNA_0071 |  |
| TB9Cs2C3 | C/D snoRNA | Tb927_09_v4 | 1629118 | 1629214 | -1 | Tb09_snoRNA_0044 |  |
| TB9Cs2C3 | C/D snoRNA | Tb927_09_v4 | 1630858 | 1630954 | -1 | Tb09_snoRNA_0053 |  |
| TB9Cs2C3 | C/D snoRNA | Tb927_09_v4 | 1625630 | 1625726 | -1 | Tb09_snoRNA_0026 |  |
| TB9Cs2C3 | C/D snoRNA | Tb927_09_v4 | 1627372 | 1627468 | -1 | Tb09_snoRNA_0035 |  |
| TB9Cs2C3 | C/D snoRNA | Tb927_09_v4 | 1623880 | 1623976 | -1 | Tb09_snoRNA_0017 |  |
| TB9Cs2C4 | C/D snoRNA | Tb927_09_v4 | 1632342 | 1632417 | -1 | Tb09_snoRNA_0061 |  |
| TB9Cs2C4 | C/D snoRNA | Tb927_09_v4 | 1634080 | 1634155 | -1 | Tb09_snoRNA_0070 |  |
| TB9Cs2C4 | C/D snoRNA | Tb927_09_v4 | 1628862 | 1628937 | -1 | Tb09_snoRNA_0043 |  |
| TB9Cs2C4 | C/D snoRNA | Tb927_09_v4 | 1630602 | 1630677 | -1 | Tb09_snoRNA_0052 |  |
| TB9Cs2C4 | C/D snoRNA | Tb927_09_v4 | 1625364 | 1625439 | -1 | Tb09_snoRNA_0025 |  |
| TB9Cs2C4 | C/D snoRNA | Tb927_09_v4 | 1627116 | 1627191 | -1 | Tb09_snoRNA_0034 |  |
| TB9Cs2C4 | C/D snoRNA | Tb927_09_v4 | 1623624 | 1623698 | -1 | Tb09_snoRNA_0016 |  |
| TB9Cs2C5 | C/D snoRNA | Tb927_09_v4 | 1633880 | 1633975 | -1 | Tb09_snoRNA_0069 |  |
| TB9Cs2C5 | C/D snoRNA | Tb927_09_v4 | 1632142 | 1632237 | -1 | Tb09_snoRNA_0060 |  |
| TB9Cs2C5 | C/D snoRNA | Tb927_09_v4 | 1628662 | 1628757 | -1 | Tb09_snoRNA_0042 |  |
| TB9Cs2C5 | C/D snoRNA | Tb927_09_v4 | 1630402 | 1630497 | -1 | Tb09_snoRNA_0051 |  |
| TB9Cs2C5 | C/D snoRNA | Tb927_09_v4 | 1625164 | 1625259 | -1 | Tb09_snoRNA_0024 |  |
| TB9Cs2C5 | C/D snoRNA | Tb927_09_v4 | 1626916 | 1627011 | -1 | Tb09_snoRNA_0033 |  |
| TB9Cs2C5 | C/D snoRNA | Tb927_09_v4 | 1623424 | 1623519 | -1 | Tb09_snoRNA_0015 |  |
| TB9Cs2C6 | C/D snoRNA | Tb927_09_v4 | 1633774 | 1633852 | -1 | Tb09_snoRNA_0068 |  |
| TB9Cs2C6 | C/D snoRNA | Tb927_09_v4 | 1628556 | 1628634 | -1 | Tb09_snoRNA_0041 |  |
| TB9Cs2C6 | C/D snoRNA | Tb927_09_v4 | 1630296 | 1630374 | -1 | Tb09_snoRNA_0050 |  |
| TB9Cs2C6 | C/D snoRNA | Tb927_09_v4 | 1632036 | 1632114 | -1 | Tb09_snoRNA_0059 |  |
| TB9Cs2C6 | C/D snoRNA | Tb927_09_v4 | 1625058 | 1625136 | -1 | Tb09_snoRNA_0023 |  |
| TB9Cs2C6 | C/D snoRNA | Tb927_09_v4 | 1626810 | 1626888 | -1 | Tb09_snoRNA_0032 |  |
| TB9Cs2C6 | C/D snoRNA | Tb927_09_v4 | 1623318 | 1623396 | -1 | Tb09_snoRNA_0014 |  |
| TB9Cs2C7 | C/D snoRNA | Tb927_09_v4 | 1633304 | 1633394 | -1 | Tb09_snoRNA_0065 |  |
| TB9Cs2C7 | C/D snoRNA | Tb927_09_v4 | 1635040 | 1635130 | -1 | Tb09_snoRNA_0074 |  |
| TB9Cs2C7 | C/D snoRNA | Tb927_09_v4 | 1629826 | 1629916 | -1 | Tb09_snoRNA_0047 |  |
| TB9Cs2C7 | C/D snoRNA | Tb927_09_v4 | 1631566 | 1631656 | -1 | Tb09_snoRNA_0056 |  |
| TB9Cs2C7 | C/D snoRNA | Tb927_09_v4 | 1624588 | 1624678 | -1 | Tb09_snoRNA_0020 |  |
| TB9Cs2C7 | C/D snoRNA | Tb927_09_v4 | 1626340 | 1626430 | -1 | Tb09_snoRNA_0029 |  |
| TB9Cs2C7 | C/D snoRNA | Tb927_09_v4 | 1628086 | 1628176 | -1 | Tb09_snoRNA_0038 |  |
| TB9Cs2C7 | C/D snoRNA | Tb927_09_v4 | 1622848 | 1622938 | -1 | Tb09_snoRNA_0011 |  |
| TB9Cs2H1 | H/ACA snoRNA | Tb927_09_v4 | 1633607 | 1633681 | -1 | Tb09_snoRNA_0067 |  |
| TB9Cs2H1 | H/ACA snoRNA | Tb927_09_v4 | 1635343 | 1635411 | -1 | Tb09_snoRNA_0076 |  |
| TB9Cs2H1 | H/ACA snoRNA | Tb927_09_v4 | 1628389 | 1628463 | -1 | Tb09_snoRNA_0040 |  |
| TB9Cs2H1 | H/ACA snoRNA | Tb927_09_v4 | 1630129 | 1630203 | -1 | Tb09_snoRNA_0049 |  |
| TB9Cs2H1 | H/ACA snoRNA | Tb927_09_v4 | 1631869 | 1631943 | -1 | Tb09_snoRNA_0058 |  |
| TB9Cs2H1 | H/ACA snoRNA | Tb927_09_v4 | 1624891 | 1624965 | -1 | Tb09_snoRNA_0022 |  |
| TB9Cs2H1 | H/ACA snoRNA | Tb927_09_v4 | 1626643 | 1626717 | -1 | Tb09_snoRNA_0031 |  |
| TB9Cs2H1 | H/ACA snoRNA | Tb927_09_v4 | 1623151 | 1623225 | -1 | Tb09_snoRNA_0013 |  |
| TB9Cs2H2 | H/ACA snoRNA | Tb927_09_v4 | 1633428 | 1633500 | -1 | Tb09_snoRNA_0066 |  |
| TB9Cs2H2 | H/ACA snoRNA | Tb927_09_v4 | 1635164 | 1635236 | -1 | Tb09_snoRNA_0075 |  |
| TB9Cs2H2 | H/ACA snoRNA | Tb927_09_v4 | 1629950 | 1630022 | -1 | Tb09_snoRNA_0048 |  |
| TB9Cs2H2 | H/ACA snoRNA | Tb927_09_v4 | 1631690 | 1631762 | -1 | Tb09_snoRNA_0057 |  |
| TB9Cs2H2 | H/ACA snoRNA | Tb927_09_v4 | 1624712 | 1624784 | -1 | Tb09_snoRNA_0021 |  |
| TB9Cs2H2 | H/ACA snoRNA | Tb927_09_v4 | 1626464 | 1626536 | -1 | Tb09_snoRNA_0030 |  |
| TB9Cs2H2 | H/ACA snoRNA | Tb927_09_v4 | 1628210 | 1628282 | -1 | Tb09_snoRNA_0039 |  |
| TB9Cs2H2 | H/ACA snoRNA | Tb927_09_v4 | 1622972 | 1623044 | -1 | Tb09_snoRNA_0012 |  |
| TB9Cs3C1 | C/D snoRNA | Tb927_09_v4 | 1866435 | 1866515 | 1 | Tb09_snoRNA_0084 |  |
| TB9Cs3C1 | C/D snoRNA | Tb927_09_v4 | 1867097 | 1867177 | 1 | Tb09_snoRNA_0089 |  |
| TB9Cs3C1 | C/D snoRNA | Tb927_09_v4 | 1865773 | 1865853 | 1 | Tb09_snoRNA_0079 |  |
| TB9Cs3C2 | C/D snoRNA | Tb927_09_v4 | 1866553 | 1866639 | 1 | Tb09_snoRNA_0085 |  |
| TB9Cs3C2 | C/D snoRNA | Tb927_09_v4 | 1867215 | 1867301 | 1 | Tb09_snoRNA_0090 |  |
| TB9Cs3C2 | C/D snoRNA | Tb927_09_v4 | 1865891 | 1865977 | 1 | Tb09_snoRNA_0080 |  |
| TB9Cs3C3 | C/D snoRNA | Tb927_09_v4 | 1866099 | 1866223 | 1 | Tb09_snoRNA_0082 |  |
| TB9Cs3C3 | C/D snoRNA | Tb927_09_v4 | 1866761 | 1866885 | 1 | Tb09_snoRNA_0087 |  |
| TB9Cs3C3 | C/D snoRNA | Tb927_09_v4 | 1867423 | 1867547 | 1 | Tb09_snoRNA_0092 |  |
| TB9Cs3H1 | H/ACA snoRNA | Tb927_09_v4 | 1866674 | 1866748 | 1 | Tb09_snoRNA_0086 |  |
| TB9Cs3H1 | H/ACA snoRNA | Tb927_09_v4 | 1867336 | 1867410 | 1 | Tb09_snoRNA_0091 |  |
| TB9Cs3H1 | H/ACA snoRNA | Tb927_09_v4 | 1866012 | 1866086 | 1 | Tb09_snoRNA_0081 |  |
| TB9Cs3H2 | H/ACA snoRNA | Tb927_09_v4 | 1866315 | 1866381 | 1 | Tb09_snoRNA_0083 |  |
| TB9Cs3H2 | H/ACA snoRNA | Tb927_09_v4 | 1866977 | 1867043 | 1 | Tb09_snoRNA_0088 |  |
| TB9Cs3H2 | H/ACA snoRNA | Tb927_09_v4 | 1867639 | 1867705 | 1 | Tb09_snoRNA_0093 |  |
| TB9Cs4C1 | C/D snoRNA | Tb927_09_v4 | 1916122 | 1916196 | 1 | Tb09_snoRNA_0094 |  |
| TB9Cs4C1 | C/D snoRNA | Tb927_09_v4 | 1919698 | 1919772 | 1 | Tb09_snoRNA_0100 |  |
| TB9Cs4C1 | C/D snoRNA | Tb927_09_v4 | 1923276 | 1923350 | 1 | Tb09_snoRNA_0106 |  |
| TB9Cs4C1 | C/D snoRNA | Tb927_09_v4 | 1930430 | 1930504 | 1 | Tb09_snoRNA_0118 |  |
| TB9Cs4C1 | C/D snoRNA | Tb927_09_v4 | 1926851 | 1926925 | 1 | Tb09_snoRNA_0112 |  |
| TB9Cs4C1 | C/D snoRNA | Tb927_09_v4 | 1934010 | 1934084 | 1 | Tb09_snoRNA_0124 |  |
| TB9Cs4C2 | C/D snoRNA | Tb927_09_v4 | 1916422 | 1916530 | 1 | Tb09_snoRNA_0096 |  |
| TB9Cs4C2 | C/D snoRNA | Tb927_09_v4 | 1923576 | 1923684 | 1 | Tb09_snoRNA_0108 |  |
| TB9Cs4C2 | C/D snoRNA | Tb927_09_v4 | 1919998 | 1920106 | 1 | Tb09_snoRNA_0102 |  |
| TB9Cs4C2 | C/D snoRNA | Tb927_09_v4 | 1927151 | 1927259 | 1 | Tb09_snoRNA_0114 |  |
| TB9Cs4C2 | C/D snoRNA | Tb927_09_v4 | 1930730 | 1930838 | 1 | Tb09_snoRNA_0120 |  |
| TB9Cs4C2 | C/D snoRNA | Tb927_09_v4 | 1934310 | 1934418 | 1 | Tb09_snoRNA_0126 |  |
| TB9Cs4C3 | C/D snoRNA | Tb927_09_v4 | 1916628 | 1916715 | 1 | Tb09_snoRNA_0097 |  |
| TB9Cs4C3 | C/D snoRNA | Tb927_09_v4 | 1920204 | 1920291 | 1 | Tb09_snoRNA_0103 |  |
| TB9Cs4C3 | C/D snoRNA | Tb927_09_v4 | 1923782 | 1923869 | 1 | Tb09_snoRNA_0109 |  |
| TB9Cs4C3 | C/D snoRNA | Tb927_09_v4 | 1927357 | 1927444 | 1 | Tb09_snoRNA_0115 |  |
| TB9Cs4C3 | C/D snoRNA | Tb927_09_v4 | 1930936 | 1931023 | 1 | Tb09_snoRNA_0121 |  |
| TB9Cs4C3 | C/D snoRNA | Tb927_09_v4 | 1934516 | 1934603 | 1 | Tb09_snoRNA_0127 |  |
| TB9Cs4H1 | H/ACA snoRNA | Tb927_09_v4 | 1919590 | 1919655 | 1 | Tb09_snoRNA_0099 |  |
| TB9Cs4H1 | H/ACA snoRNA | Tb927_09_v4 | 1923168 | 1923233 | 1 | Tb09_snoRNA_0105 |  |
| TB9Cs4H1 | H/ACA snoRNA | Tb927_09_v4 | 1926743 | 1926808 | 1 | Tb09_snoRNA_0111 |  |
| TB9Cs4H1 | H/ACA snoRNA | Tb927_09_v4 | 1930322 | 1930387 | 1 | Tb09_snoRNA_0117 |  |
| TB9Cs4H1 | H/ACA snoRNA | Tb927_09_v4 | 1933902 | 1933967 | 1 | Tb09_snoRNA_0123 |  |
| TB9Cs4H2 | H/ACA snoRNA | Tb927_09_v4 | 1916298 | 1916370 | 1 | Tb09_snoRNA_0095 |  |
| TB9Cs4H2 | H/ACA snoRNA | Tb927_09_v4 | 1923452 | 1923524 | 1 | Tb09_snoRNA_0107 |  |
| TB9Cs4H2 | H/ACA snoRNA | Tb927_09_v4 | 1919874 | 1919944 | 1 | Tb09_snoRNA_0101 |  |
| TB9Cs4H2 | H/ACA snoRNA | Tb927_09_v4 | 1930606 | 1930676 | 1 | Tb09_snoRNA_0119 |  |
| TB9Cs4H2 | H/ACA snoRNA | Tb927_09_v4 | 1927027 | 1927097 | 1 | Tb09_snoRNA_0113 |  |
| TB9Cs4H2 | H/ACA snoRNA | Tb927_09_v4 | 1934186 | 1934256 | 1 | Tb09_snoRNA_0125 |  |
| TB9Cs4H3 | H/ACA snoRNA | Tb927_09_v4 | 1917164 | 1917219 | 1 | Tb09_snoRNA_0098 |  |
| TB9Cs4H3 | H/ACA snoRNA | Tb927_09_v4 | 1920740 | 1920795 | 1 | Tb09_snoRNA_0104 |  |
| TB9Cs4H3 | H/ACA snoRNA | Tb927_09_v4 | 1924318 | 1924373 | 1 | Tb09_snoRNA_0110 |  |
| TB9Cs4H3 | H/ACA snoRNA | Tb927_09_v4 | 1927893 | 1927948 | 1 | Tb09_snoRNA_0116 |  |
| TB9Cs4H3 | H/ACA snoRNA | Tb927_09_v4 | 1931472 | 1931527 | 1 | Tb09_snoRNA_0122 |  |
| TB9Cs5C1 | C/D snoRNA | Tb927_09_v4 | 1822194 | 1822293 | 1 | Tb09_snoRNA_0077 |  |
| TB9Cs5C2 | C/D snoRNA | Tb927_09_v4 | 1822681 | 1822770 | 1 | Tb09_snoRNA_0078 |  |
