# Supplementary material for: A comparative genome-wide study of ncRNAs in trypanosomatids
Source: BMC Genomics. 2010 Nov 4;11:615. doi: 10.1186/1471-2164-11-615 (PMC3091756; doi:10.1186/1471-2164-11-615)
Supplement: Additional file 2 — List of missing/mis-annoted ncRNA. List of the additional RNA genes that have been reported previously in the literature, but have not yet been incorporated or are misannotated in the GeneDB genome annotation. [file 1471-2164-11-615-S2.DOC]

| **Name** | **Type** | **Chromosome** | **Start** | **End** | **Strand** | **Reference** |
| --- | --- | --- | --- | --- | --- | --- |
| MRP | misc RNA | Tb927_10_v4 | 1729478 | 1729998 | 1 | Barth et al. 2008 |
|  |  | Tb927_10_v4 | 1739213 | 1739733 | 1 |  |
| tRNA-Selenocysteine | tRNA | Tb927_09_v4 | 505605 | 505697 | 1 | Cassago et al. 2006 |
| sRNA-76 | tRNA-like | Tb927_04_v4 | 1287302 | 1287374 | -1 | Beja et al. 1993 |
| SnR30 | C/D snoRNA | Tb927_11_01_v4 | 2702565 | 2702732 | 1 | Barth et al. 2005 |
|  |  | Tb927_11_01_v4 | 2703579 | 2703653 | 1 |  |
| TB3Cs2H1 | H/ACA snoRNA | Tb927_03_v4 | 1159820 | 1159887 | -1 | Myslyuk et al. 2008 |
| TB3Cs2H2 | H/ACA snoRNA | Tb927_03_v4 | 1159573 | 1159656 | -1 | Myslyuk et al. 2008 |
| TB8Cs4C1 | C/D snoRNA | Tb927_08_v4 | 512761 | 512850 | 1 | Myslyuk et al. 2008 |
| TB8Cs4H1 | H/ACA snoRNA | Tb927_08_v4 | 512961 | 513030 | 1 | Myslyuk et al. 2008 |
| TB8Cs4H2 | H/ACA snoRNA | Tb927_08_v4 | 513208 | 513280 | 1 | Myslyuk et al. 2008 |
| TB9Cs6H1 | H/ACA snoRNA | Tb927_09_v4 | 1029060 | 1029140 | 1 | Myslyuk et al. 2008 |
| TB9Cs6H2 | H/ACA snoRNA | Tb927_09_v4 | 1029329 | 1029396 | 1 | Myslyuk et al. 2008 |
| TB10Cs5H1 | H/ACA snoRNA | Tb927_10_v4 | 2180028 | 2180113 | 1 | Myslyuk et al. 2008 |
| TB10Cs5H2 | H/ACA snoRNA | Tb927_10_v4 | 2180159 | 2180228 | 1 | Myslyuk et al. 2008 |
| TB10Cs5C1 | C/D snoRNA | Tb927_10_v4 | 2180341 | 2180429 | 1 | Myslyuk et al. 2008 |
| TB10Cs5H3 | H/ACA snoRNA | Tb927_10_v4 | 2180510 | 2180580 | 1 | Myslyuk et al. 2008 |
| TB11Cs5C1 | C/D snoRNA | Tb927_11_01_v4 | 4208781 | 4208868 | -1 | Myslyuk et al. 2008 |
| TB11Cs5H1 | H/ACA snoRNA | Tb927_11_01_v4 | 4208681 | 4208753 | -1 | Myslyuk et al. 2008 |
|  |  | Tb927_11_01_v4 | 4208288 | 4208360 | -1 |  |
| TB11Cs5H2 | H/ACA snoRNA | Tb927_11_01_v4 | 4208207 | 4208278 | -1 | Myslyuk et al. 2008 |
| TB11Cs5H3 | H/ACA snoRNA | Tb927_11_01_v4 | 4208430 | 4208500 | -1 | Myslyuk et al. 2008 |
| TB11Cs5C2 | C/D snoRNA | Tb927_11_01_v4 | 4208509 | 4208588 | -1 | Myslyuk et al. 2008 |
| TB10Cs2'C1 | C/D snoRNA | Tb927_10_v4 | 2873115 | 2873202 | 1 | Barth et al. 2008 |
| TB3Cs1C-1 | C/D snoRNA | Tb927_03_v4 | 255623 | 255706 | 1 | Barth et al. 2008 |
|  |  | Tb927_03_v4 | 371910 | 371993 | 1 |  |
|  |  | Tb927_03_v4 | 651333 | 651413 | -1 |  |
| TB7Cs1C1 | C/D snoRNA | Tb927_07_v4 | 1060072 | 1060158 | 1 | Barth et al. 2008 |
| TB9Cs1'H1 | H/ACA snoRNA | Tb927_09_v4 | 568015 | 568083 | -1 | Doniger et al. 2009 |
|  |  | Tb927_09_v4 | 568117 | 568187 | -1 |  |
| TB10Cs4'H1 | H/ACA snoRNA | Tb927_10_v4 | 1824036 | 1824135 | -1 | Doniger et al. 2009 |
| U5 | snRNA | Tb927_10_v4 | 1907326 | 1907400 | -1 | Dungan et al. 1996 |
