# Supplementary material for: A comparative genome-wide study of ncRNAs in trypanosomatids
Source: BMC Genomics. 2010 Nov 4;11:615. doi: 10.1186/1471-2164-11-615 (PMC3091756; doi:10.1186/1471-2164-11-615)
Supplement: Additional file 3 — Complete list of candidate ncRNA. The complete list of all the sequences of the 72 ncRNA candidates conserved in four of the six genomes. The first 29 were conserved in all of the six genomes. [file 1471-2164-11-615-S3.DOC]

| **Candidate #** | **Chrom** | **Start** | **End** | **Sequence** | **Comments** |
| --- | --- | --- | --- | --- | --- |
| 1 | 1 | 296412 | 296540 | TTTCTTCGGTTGAGGCATTATCGTTGGCGGAGATCGAAGTAGGTTAACATACGGATACAA  GTGCGAGTGAGCTGAAGTGGCTATGTTTCGCTCGTCGATGGTAGTGCGGTCCTTTACGCA  GGAATCTTTTTATCGCATGAGCAAGTACATCAAGAGCCGCAACCCCAACGAGAGGTACCT  GCGCACTGGCCACATTGTACTTGAAACGCTAAAGAGGTACCATTCGTATGTGTTGGCCCT  GGTATTTACTCTGGGGGTCACGTGCTACGACCAAATCAAGCACCCAGAGAAAATTAGAGT  ACCCGTACACGATGGTCGATCACTTGTTC | ExpCandidate #32 |
| 4 | 9 | 350252 | 350344 | TGCTGAGGGGTCTGCACAAGTTCTCCCTCGTATGTTTTTAGCTCGCTGTCAGATCCCTCG  TAGGAACAAACCAAAACACAAACCCGAGTGGGT |  |
| 5 | 11_01 | 4144018 | 4144091 | TCATCAAGTATATCCTTCGCAAGCCGCCACCCCCACGGGTTCTCTGCACACGGGTATGGT  GGGGCGAGGAGGAG | ExpCandidate #31 |
| 7 | 9 | 509442 | 509534 | ACACCACAAAGGCCGAATCGAACGGCTGCGAGTCCAACTCGCCTACGGGTTTGAAGCCCG  CACCCAGCACCAGCTGAGCTCATCGTGGCGCAA | ExpCandidate #2 (tRNA-sec) |
|  |  | 505605 | 505697 |  |  |
| 9 | 9 | 1370363 | 1370433 | CGTCTTGGCCACCAACTCATTAAGGAGCCATTGGGTCATGAGCAAACTACTTTCGCCCGCGGCCAGCGTACGATGGCAAATACGCTTAAGGGCTTTTAC | ExpCandidate #9 |
| 10 | 10 | 698209 | 698350 | GAAACGAAAGGTGGCGATTATATTCGGCTACATTGGCGAGCGGTACTGTGGACTGCAATG  GAACCACCTACCGAATTACCCAACAGTTGAGGAGGAATTACTGCGTGCTCTGCACCGTGC  TGACATGATATCTGAGGAGAAC |  |
| 12 | 11_01 | 704251 | 704301 | TTTGGATTTGCCTGAAACCATTGTCCCTTCATGGGTTCCTCAATTACAACA | ExpCandidate #33 |
| 14 | 4 | 671279 | 671323 | ATGTCACTGTGGCCGTTTCGTACGGGCAATGACGAACGGTTTCAA |  |
| 15 | 11_01 | 2644054 | 2644272 | ACCATGGCGAAGTCGAAGAACCACACCAATCACAACCAGTCGCGGAAGAACCACCGCAAT  GGGATTAAACCCCCGCTGCCGTTGTACATGTACAACTCGAAGCGCGGTGGGTGGCTGCCG  GCCCTCGTTAACACCCGACGTGTGAGGAAGAACAATCAGAAGGCTGCGCTGAAGGCGCGC  CGTGAGCGACTCGCCGCCCATCAGGCCGCACAGAAATAA |  |
| 17 | 11_01 | 1986605 | 1986679 | TCTCCGTCGACCTCAGCACCCCAAATGGCCGCAAGCTCAATTTGTCGCTCCTTCTCCAAA  TCACGCTTCCCCATC |  |
| 18 | 8 | 1763482 | 1763627 | ATGGGGACCATCAGCCGCCCTGGAGGGATGCGGCCCAAGTGGCACAAGAAGCGCATAAAGCGCTTAAAGCGCCGACGACGACGCATGCGCCAGCGCTCCAAGTAAGCGCCCTGATGCATC | ExpCandidate #1 |
| 19 | 11_01 | 4478330 | 4478489 | ATCATTTCCCTTTCCCTCTGCTTTTCGAAAAAAATAATCCAACTGGCAGGGCATGCAGCACTCAACAAGTTCAACTGTTCAGCGCACTTCTTTTCCGGCATCTCGGGGAGCGAATCAAGACATCTGTGGTATGAGTCTCGAGCTTCGTAGCATAATTCTCTCTTGTGTGCGTTTTGCAT | ExpCandidate #6 |
| 20 | 10 | 698362 | 698463 | AAAGGTTCAGCAGAAACTTAACTGGGAGCGTGCCAGTCGCACCGACAAGGGTGTTCACGC  CCTGCGCAATGTGATCTCGCTTAACGTGATGCTACCGTATGC |  |
| 21 | 3 | 1379222 | 1379301 | GAAGGACTTCGTCACGTGTATCACGGATTTAATCTTTTCTTCTTGAATACGCGTCTGGCT  TGGAAATACCTCCCGGCAGC |  |
| 23 | 3 | 1147610 | 1147724 | TACGTCTCCCGCAGATGTGTCGCAAAAACGGAGAATCCGTAAGTCGATGATACAACAAGA  CCAGCATAAACTCCTGCCATCAGCATGCGTACTCGCGCAAGGTCATCGACAAGGT |  |
| 26 | 8 | 2235539 | 2235625 | GACGCCGGCCACCTCAACGTGGTGCCAAGTTCCAGCGCCCCGTATTTTCAGAGGAGGCCAATAGTCAGCAGCGTTTCTCT  AGTGGGG | ExpCandidate #23 |
| 27 | 11 | 37888 | 38001 | AACGTTAAACGTCCCTATCGTATAGAAATACGTATGGAACACGACAAAAAGCGGCGGATGAAACGACGCAACATTGGCTGCCGGCGCATGATGAAATCGT | ExpCandidate #3 |
| 28 | 10 | 1679504 | 1679598 | ATGATTACAAAGCAATTTTATCCTCACCCTGACGTGAGTGGTTCTGTGACGAGAAACCGATTGACAATAATACCAAGATCTGCTATGA | ExpCandidate #5 |
| 29 | 10 | 1681621 | 1681689 | AGACGCTGTATTAATCCCGCCCCGTATGGCAAACTCATGCCCGGGGCGATTCATGCACAGCGGAGATGC | ExpCandidate #51 |
| 31 | 11_01 | 163062 | 163301 | CTAATACCACCGCCACCGTTCTAGACGCTTGTATTTATGAATAAACTCCTTTGTGTACAC  CTGTGACGCGTAGTAGTTCTTGTCCTGGAAGTGCGAGTAGTGCGGCATGTATGCTGCATG  ATACAAATACACGATAATCGCCATTGGGGTAAACCCAATAAAGAAAAACGGCAGAAACGT  GTCGGTCATTGTCCATTGATGTGGGGGATTAAACCAACGTCCGGAAAAGGGTGGTGCGAA | ExpCandidate #30 |
| 34 | 10 | 3478086 | 3478182 | GCCTCATCCATTGTCATAAACTCAGTTTGGAAAAGCGAATTAAGTAGCGTTGACTTACCGCTGCTCTGCCCGCCAAACAC  ACCAACCACATGATAA | ExpCandidate #19 |
| 35 | 11_01 | 4446474 | 4446564 | ATACTCTTCATGAGCACGTGGGGGGTCGATCAACTCCAGAGCCATAGCGAGGTTGTGGTG  CGCCACTGCTAGCAAAGAAGGGTCCTCAGGA |  |
| 37 | 11_01 | 1986209 | 1986399 | TGGTTGGTGCCGTCCAGCACCTTCACGGCGTGCAGCGCCATCGCCTTGTTACGAAAAGTC  AGCACCGCAAAATGTGTCTTGTTCAGTAAACTACCCTGCTGAGATGTCCTCAATTCCACT  TTCGGCTCATAAGCCTCAAAAACGGTGCGAACATCGTTTCGTTCAGTTCCAGGTCCAACA  AAGCAGTGCAA | ExpCandidate #34 |
| 42 | 11_01 | 4446652 | 4446800 | AGGAACGCTACGGGGGATTGTTGCCCCGTGCATTGCATCGATTGCTGCATGTAGTCCAGG  GCAGTACTAAAATGCCCCCGGTGGCGCTCCATGCAACCGTGGTTGTTCAGGGTCACACCG  CGCAGTCGTCGGCGGCGCTCATCGTCTTT |  |
| 46 | 10 | 1999051 | 1999166 | GTTAACTGCTCAACTGCCGCTGCGCTCCCATCGGACTCGTACGTTTTCAGCAACTCATCA  GAGAAAACATCAGAGAAATGCTGCATATAATCGTCTCTAGCCTTCTGCACAGCTGA | ExpCandidate #29 |
| 47 | 11_01 | 2238304 | 2238490 | GATGTCGCAAAAAGGTTTGAAGGGTAAAACTCGCGGGAAGAGCGAAGGTGCCTTGCGCAA  GTACGTGGGTGAGGCCAAGAAGAAAACAGTCTTCAACAAAGGGCGTAAGACATTACAAAC  CAAAGCGAGGGCAAACTACATATCGGCTGTAGAGTCACATATGGCTTCCCGTGTTCCATC  TGATCAG |  |
| 48 | 11_01 | 2238507 | 2238597 | TTGTGAAGGCAACTGGAGGATTGCAGCCGAAAAAGAAGCATATGAAGAAGCCACTGACTA  GAGGGCGTAAGCGTAAGGGCGACAAGAAGGG |  |
| 49 | 11_01 | 616706 | 616835 | ATGCTTCTAGGAGGTTTTGTTCCCCGCCGCTTCTCGCAGTTTAACCGCGACCCGTGTTGGATGTTCTTCATATTTTCTGTCGGCTTCTGGCTGGGCGAATATCCGGCGATGATGATCAAATATAATGCCC | ExpCandidate #4 |
| 50 | 3 | 981626 | 981680 | AGATGGGGTTATCAATGCGGACGAGTACATTTACATGTGGCGTGCCCTCCTTGCC |  |
| 53 | 9 | 1380620 | 1380769 | TAGGAAGCTTACACTTCGCAATGACCACTGCCCTCGCTGCGACCAGTTGACTCTGTTCCGATTCATGAGTGTGAGTGGGATGGTTGGAAATATGCCATTCAAACCCATCGGTGTCCCGGGTCCCTCGTATGCCACACTATGGTGGCGTAA | ExpCandidate #8 |
| 57 | 1 | 322289 | 322431 | CAGTGCGTCTGCGCAAGTTGCTTCTCACCGCTTCCGTTACCTCGTCCCGACGCAACAGGAACACCGTTTCAATGCCCCCAATGCCGAATGGCTGGTATAGATATGAAAGTGCTTTACACCCGTCGTGCGTTCCCAACAGAG | ExpCandidate #7 |
| 58 | 6 | 1342102 | 1342185 | TGATGCCGTACCCATTGGGGTCGTCTCTTTGGGTCAAGTGCGACGACGGAGAGTGGTGGCCCGCTACCGTCCGTGAAGTGGAGACTGAG | ExpCandidate #10 |
| 59 | 11_01 | 2528884 | 2528963 | TGACGAAGCAATTACGAAATGTAGTGAACCAATGAATCCCCTCACCCGAGGAAAAGAAGACGTAAAAACACATGCAACAGCTAA | ExpCandidate #11 |
| 60 | 9 | 314882 | 314946 | GTGCTGGGAGCTGGGCCGCCGGCCTGCAGCTATCGCGCGGAATGTGTGGCGATGGAGGCGGGGTT | ExpCandidate #12 |
| 61 | 10 | 1968139 | 1968305 | TTATCGCTCACTGGTACTGCGGGCACAAGTTTCGCCACCGCTTCATGCGGGACAAGCGGTTTCATCCGTCGCTACAGGCGTCCCACGACGCCAGGAATCGTTTTAGTAAGCGAAGACACTTCAAAACGAACAGGTGGAACTACCAGCAGGCGTACCGTGACATGCCGT | ExpCandidate #13 |
| 62 | 2 | 300622 | 300695 | AATTTCGCGCTTAGGGAGTCCATTGCGATGATGAATAGCAACGACCCCAGCACGGAACCTTGTGGAACACCACACGTGA | ExpCandidate #14 |
| 63 | 4 | 1488062 | 1488091 | TCCAGTGCCCATACTGCCCAATGAATTGCA | ExpCandidate #15 |
| 64 | 1 | 255249 | 255278 | TCGGCGGCCTCTCAGTCGTCTTCGTCGTCGT | ExpCandidate #16 |
| 65 | 11_01 | 2031239 | 2031295 | TGTGCGCGTGGGGGAGGGGAAGAGGGAAGGGGAGTGAAGGTGCCATCCCCGATGCGAT | ExpCandidate #17 |
| 66 | 10 | 1525377 | 1525459 | CGTTGGGATGGGAAAAGGGGAGGGGATGGATATTTGCGGTAGTTTTATTGCGCACAGTTGCTGTTACACACCAACTGATGTGT | ExpCandidate #18 |
| 67 | 10 | 2199386 | 2199537 | ATGATTTACGACATTACCACTCCGCAGTACCAGCAGTTCCTCCGCTCATGCGGCCGCCGCCGTGAGGATTACGTGAAGGGATCGTCCACAGGTTTCTCGGGTAACAAGCAGACCACGAAGCCGGCGGGAGCTGCAAGTACTGGGCTGTAGCG | ExpCandidate #20 |
| 68 | 9 | 2596449 | 2596527 | TACAGACATGTTATCTTTATTAATGGCACTTAAAACGGATCCTACAGAAGTGACAGATACCATGCTACGACGCATTTAGG | ExpCandidate #21 |
| 69 | 2 | 584827 | 584900 | TCTGCATTGGAACGTATGGCATCATGGCGGATTGGGTGTATCGCGGTTATGTGGATTTGTGGTATGGCGTGTAT | ExpCandidate #22 |
| 70 | 10 | 4039096 | 4039161 | CCTTATTAATGGAGCTCAAAACAGGTCCTGCAGCAGTGACTGATTTTATTCTACGACGCATATGGG | ExpCandidate #24 |
| 71 | 11_01 | 2617841 | 2617922 | TG CCGGACGATG ATGGCATCAG TGCAGAGTTGCGGGGCTTTG GCGAGTATGG CGGGTCTATT TCCGCGTTTG TTAAAGCAGG | ExpCandidate #25 |
| 72 | 11_01 | 3464659 | 3464791 | ACACCCGTTGGCGAAACTTTGTGACCTCCCGACAAACTGGGCACTGGGATAAATTTGATGCACAAGAAACACACAAACAGTAGTGTCCACATGGTACCAGCACCGTGTCCTTTGGGCCAAAGCAAATGACACA | ExpCandidate #26 |
| 73 | 7 | 535059 | 535139 | AACAAAGACGCGGAGAAATTCTCCACTGAATTCCATACCATTCATATTACCCGCTGCAGCAATCGCATCATCCGCCTCTTCA | ExpCandidate #27 |
| 74 | 10 | 2445794 | 2445921 | TGCACCGCCTATCCCTATCTGCCCCTCTGTTCCGTTTGCCTCAATCCCTGTAAGTATTGCTTGGGAACTAATAGGTGGTGCCCTCACTGCTATGAGAAGCGCTGCATCAATGTGTATAAGCGGGTTGT | ExpCandidate #28 |
| 75 | 7 | 1203152 | 1203250 | CGGCTTCGTTTTCCACGAATCGGAAGAGCCAAAGGGACTTCTTTGTCCCCCAAAGTTGCTTCCAATAAAATCTTTCGCGCTGGAGCGTCTAGAACATTT | ExpCandidate #35 |
| 76 | 11 | 4257881 | 4257959 | ACACAATGGTCTGAGTAGCGTGTTGGGTCGTTGTGTACGTTGTCGTCGCCGTATTCAGGTGGATTTGGAGCTTTT | ExpCandidate #36 |
| 77 | 10 | 940801 | 940901 | CAAATAGAGCTGCAGTTCCCGCAACGCCTTCGACTCCTGCCGGGGGGTTCACAGTCCATAAAAGTCCTGAAATGCGACCGTGTGGTACCAGAATGCGGCCAAGA | ExpCandidate #37 |
| 78 | 9 | 2923952 | 2924034 | CCCTGGTGACGCCGGCAACCTCATCGTGGCGCCAGGCTCTAGTGCTTCGTTCACGAGGAAGTCAAGTGCCTGCATTATACCCA | ExpCandidate #38 |
| 79 | 11 | 3236031 | 3236098 | ACGTATGGAGGGTAATTTCTACGTTTCTGACTTTGTGATCAAACGGGTCCTGCGCAGCAATTACGGCC | ExpCandidate #39 |
| 80 | 10 | 2629376 | 2629451 | CTTTACGAGGTGAATGTGACACACAAAAACCGCATTCCTTCCCCTAACGTTAGCAACCATCTTGAGTACGAACTAC | ExpCandidate #40 |
| 81 | 10 | 3735051 | 3735195 | ATGAACTTGGGTCAAGTGCCCCGCCCAAGGGATGAGCCCGAGCCCTTTAACGATTTTGAGCCCCGTCGCAAGTGCTCCATATGCTGCGGTCTTCGGTGCGCTGCTGTAACTGGAGGTGCACTCTTTCTGATCGTGCTCCCATGTA | ExpCandidate #41 |
| 82 | 10 | 3227616 | 3227701 | TCGTGGACTAGAGGAGGTGGTGAAAGTGCGGCCCGCCGATCCACTCGCATTTTTGGCAGCGTACCTGCTGAGCAACAATCCGCAGA | ExpCandidate #42 |
| 83 | 8 | 169552 | 169701 | GACAAGAATCGTGTCAACCAGGGTCGACGCGAGGCTAAAATGCGCGCCCAAGGAGCAAAGCACCACACAGTTTCAGCCAAGGAATCAATCGAAATAAAGCGCGCACATAAGAAAAACACCCAGGCCATAGCGATGGAACTGCGTGCAAAG | ExpCandidate #43 |
| 84 | 2 | 903902 | 904001 | GGGCGGTGCCCTTCACGGTAGTTGAAGAGAAGCCAACGGGTTGTCGAGGGCGAATCATTACATGGCCAAAGTCTAAGAATGCACAGGACGATTATGAAGC | ExpCandidate #44 |
| 85 | 5 | 1336410 | 1336493 | GGGTAGAATGCGGGCTATTGGCTTCCTTCTGGACGGAGCACTAGACGCTGGCACCATGATGAGATGGGCGGTGTAGGCAGGGAC | ExpCandidate #45 |
| 86 | 10 | 2782008 | 2782050 | GTACGAAAGCAACCCACCGCGTCGTCCCAACATAATCACTGTG | ExpCandidate #46 |
| 87 | 10 | 2738452 | 2738540 | AACCCATTTGAAGGCCTCCCTCCCACTCCCCGCAGCGAGGAGGACGATATCCGGAAGGGTATATTCGGTCGGGTGTTGCGTCTACTTCG | ExpCandidate #47 |
| 88 | 9 | 1664677 | 1664744 | TTAGCTCTGGCCCAGAGCGCAACGGATGTGGGGAATGTAGGCGCTGCACAAGAATTCTACGAGGTCGC | ExpCandidate #48 |
| 89 | 2 | 136955 | 137000 | CGTGCTCACTGGTGTTTTCTTTACTAAAATGCACATCCATTTTTGTG | ExpCandidate #49 |
|  |  | 171104 | 171149 |  |  |
| 90 | 10 | 698362 | 698463 | GGTAGAATGCGGGCTATTGGCTTCCTTCTGGACGGAGCACTAGACGCTGGCACCATGATGAGATGGGCGGTGTAGGCAGGGAC | ExpCandidate #50 |
| 95 | 9 | 1249202 | 1249287 | TGCGGTCGAAGAGCATATGTGAAATGCCGTGGCCAATGACGGAACCGGCTGCAACTGATG  CCACATTTCCAAGGAGACCGCTACCC |  |
| 96 | 5 | 62863 | 62898 | AGTAGCGTTAGGTCATCTGCAAAGAATCCGTGCTGC |  |
| 97 | 11_01 | 2097117 | 2097244 | TCACCGCAGCCATGCACTTCTGGGAGCAGGTGAGACGTACACATGCGCTGGCTGCAATGC  TCACAGGCCGGCAGCGCGGTATATGTCAGGTTTGAGCACCCTTTGAATGCACAAACAACG  GACATATT |  |
| 99 | 2 | 136955 | 137000 | CGTGCTCACTGGTGTTTTCTTTACTAAAATGCACATCCATTTTTGT |  |
| 111 | 10 | 1701006 | 1701100 | TGTTTGGAGAGTTTGATGGAATACTTCAACACATACGCATTTGTTCATGTTGCCATCTAT  GGTTGTGGGTACACTGAAGCAGCGAAAATGACATG |  |
| 112 | 10 | 2736706 | 2736800 | TGGTAGGGCTACCCAGTACATTGGCATTTGAATCCATACTAAGGGAGTGCCCTTTAAGCA  CACGTGTTATTCTCGTAGAAGAGACTGACAAGGAT |  |
| 117 | 10 | 43058 | 43097 | AGTAGCGTCAGGTCGTCTGCAAAGAATCCGTGCTGCAGTA |  |
| 118 | 10 | 15901 | 15951 | TCGCAAGTAGCGTCAGGTCGTCTGCAAAGAATCCGTGCTGCAGTAACGGCA |  |
| 123 | 10 | 98001 | 98049 | TTGCTCGCGTGCCTTCTATTAATTTAATGTTTGTATACATATGTATCTG |  |
| 124 | 2 | 171101 | 171149 | CTACTTGCTCACTGGTTTTTTCTTTACTAAAATGCACATCCATTTTTGT |  |
| 125 | 10 | 2782008 | 2782050 | GTACGAAAGCAACCCACCGCGTCGTCCCAACATAATCACTGTG |  |
| 126 | 9 | 2484908 | 2484948 | CAACAGCTTTAACTTCGGTGCATTCGGACTTGACGATCAGT |  |
