# Supplementary figures and images for: A comparative genome-wide study of ncRNAs in trypanosomatids
Source: BMC Genomics. 2010 Nov 4;11:615. doi: 10.1186/1471-2164-11-615 (PMC3091756; doi:10.1186/1471-2164-11-615)

## Slide 1
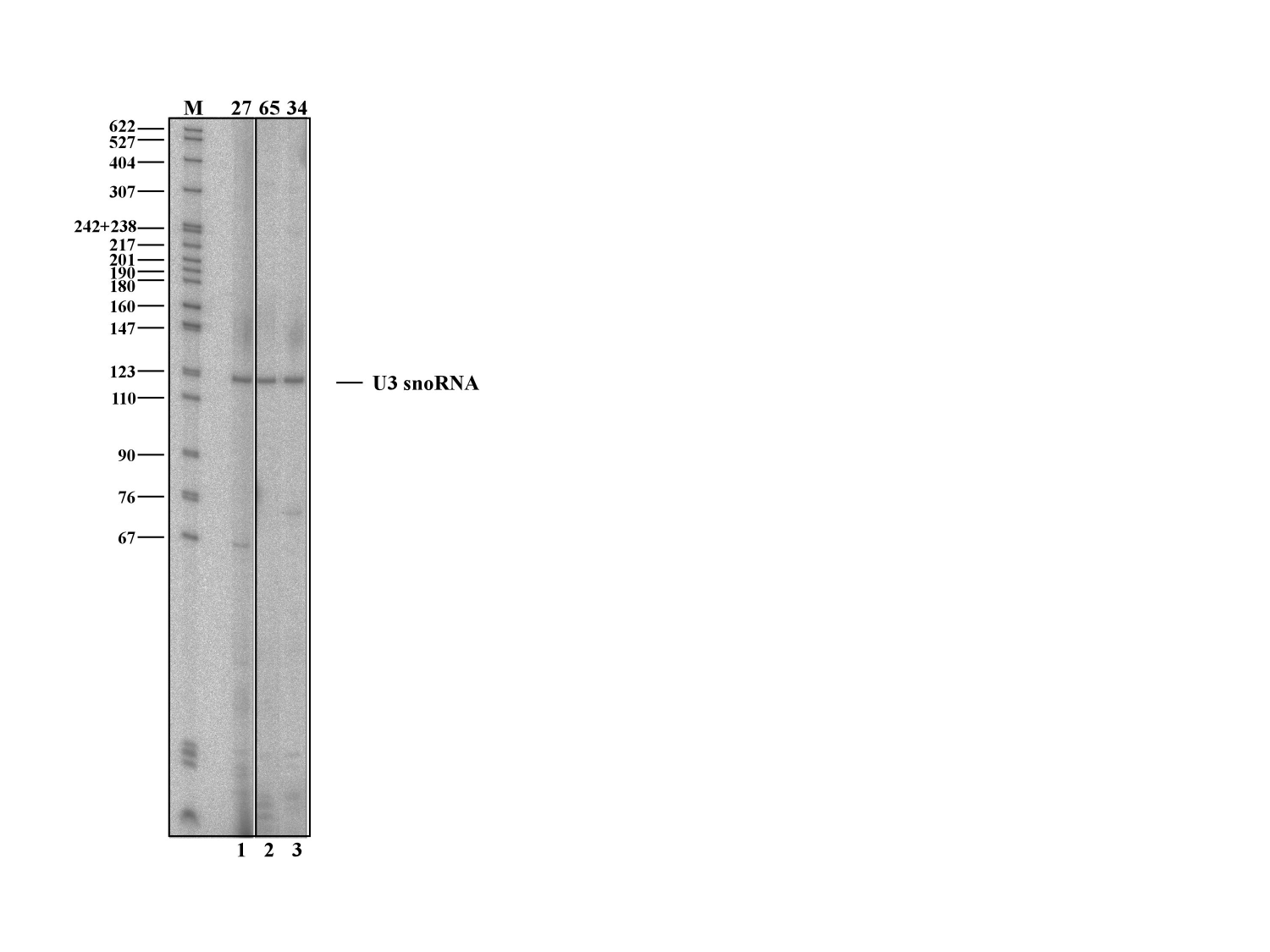

Supplement: Additional file 5 — Results of expressed candidates in primer extension assay with an internal control. Primer extension was performed as in Figure 2 with the addition of an internal control to each sample. The primer extension reactions contained a primer specific for the candidate as well as a primer specific to U3 snoRNA. [file 1471-2164-11-615-S5.PPT]

## Slide 1
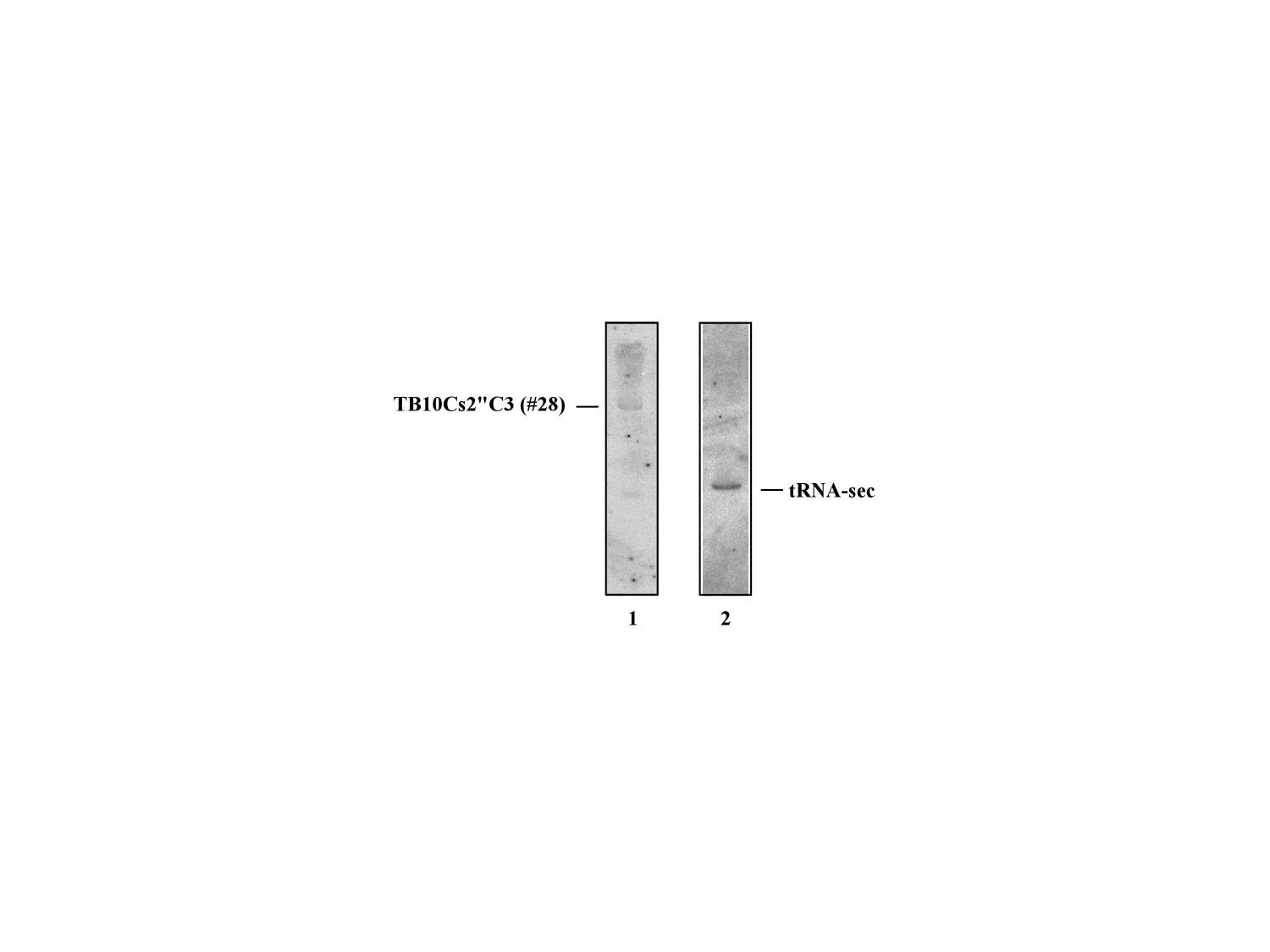

Supplement: Additional file 6 — Northern blot analysis of two of the candidates. RNA was prepared from PS cells, separated on a 10% denaturing polyacrylamide gel, and subjected to Northern analysis with the indicated oligonucleotide anti-sense probes. [file 1471-2164-11-615-S6.PPT]

## Slide 1
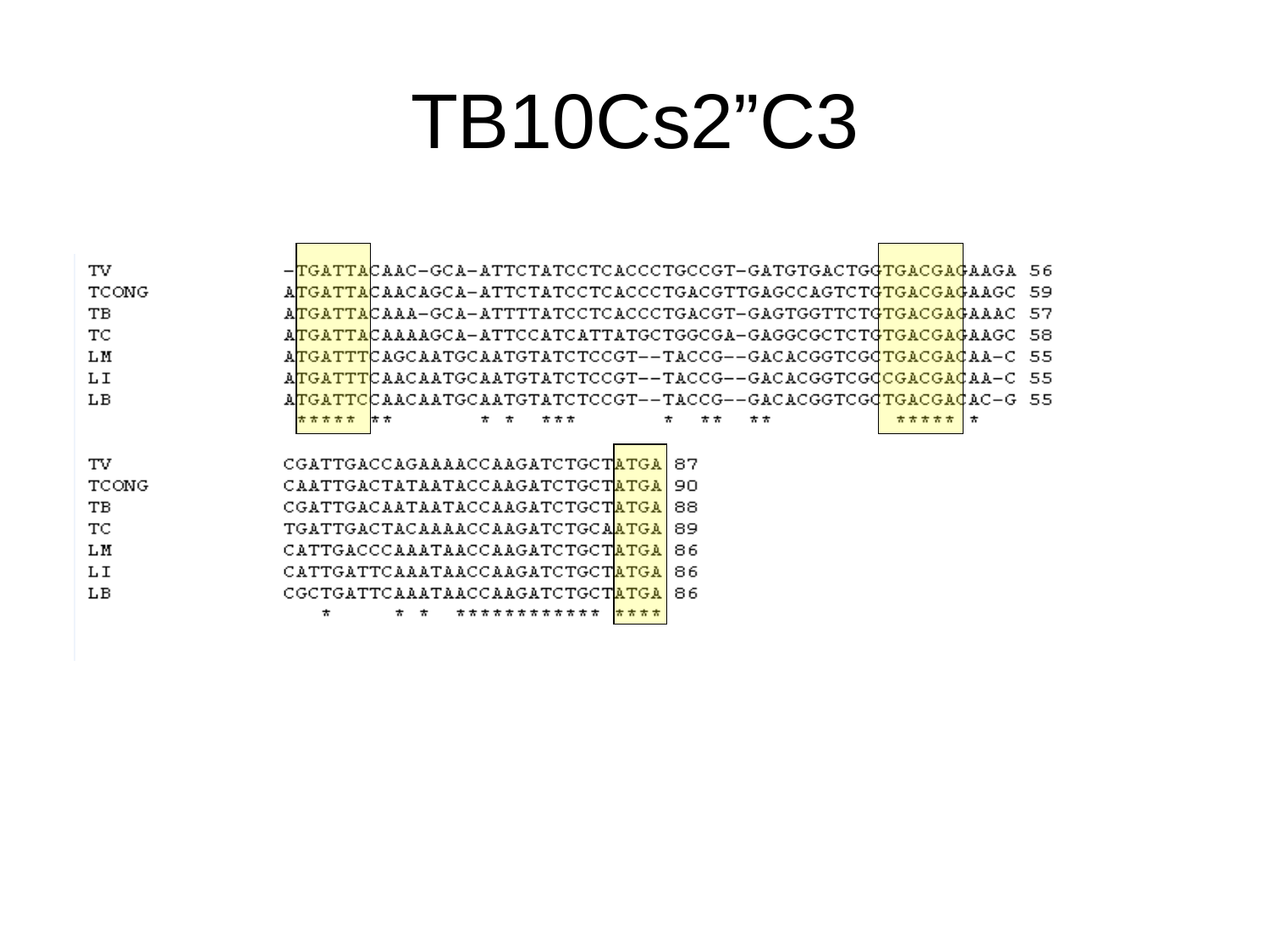

# TB10Cs2”C3

## Slide 2
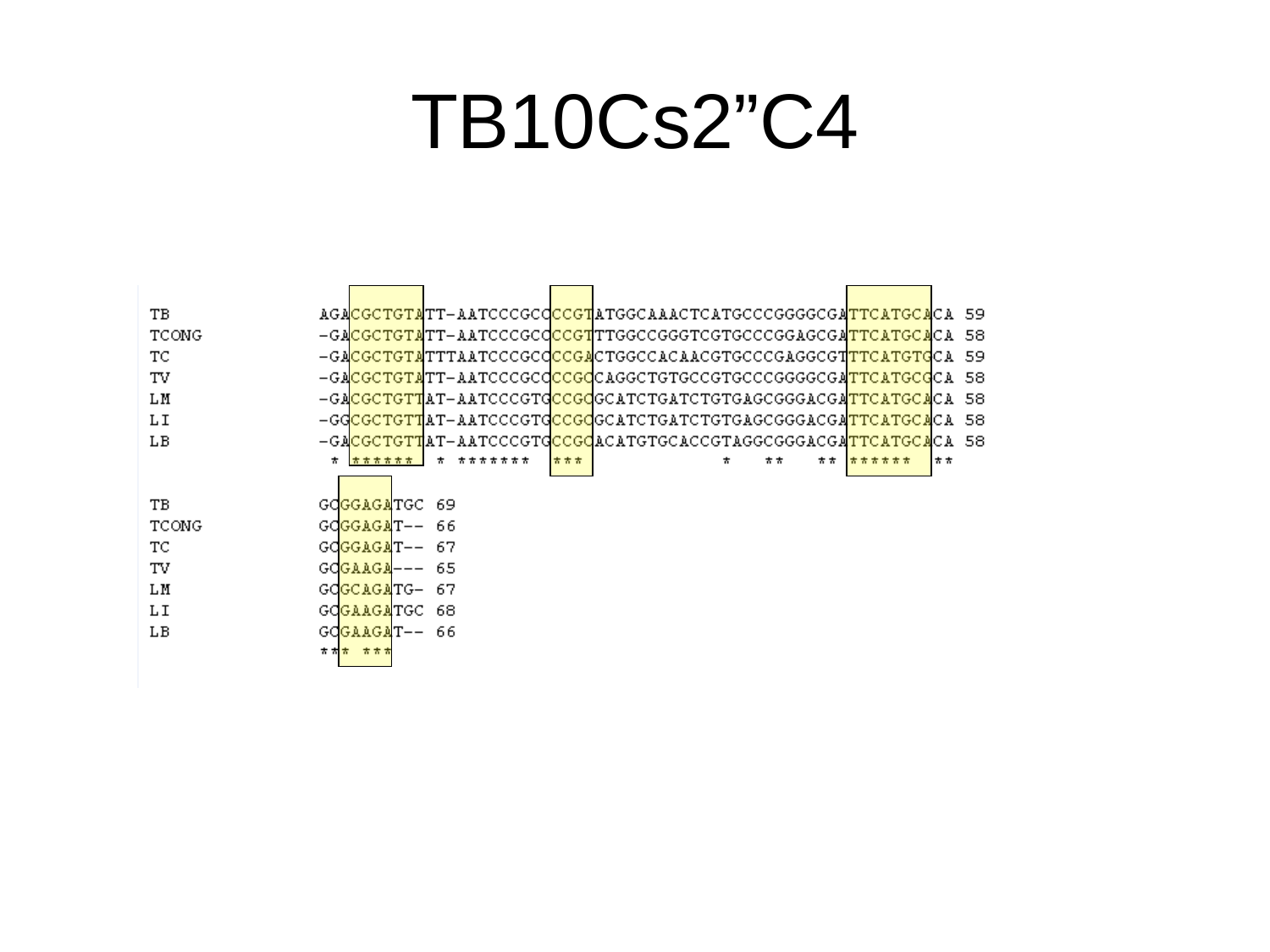

# TB10Cs2”C4

## Slide 3
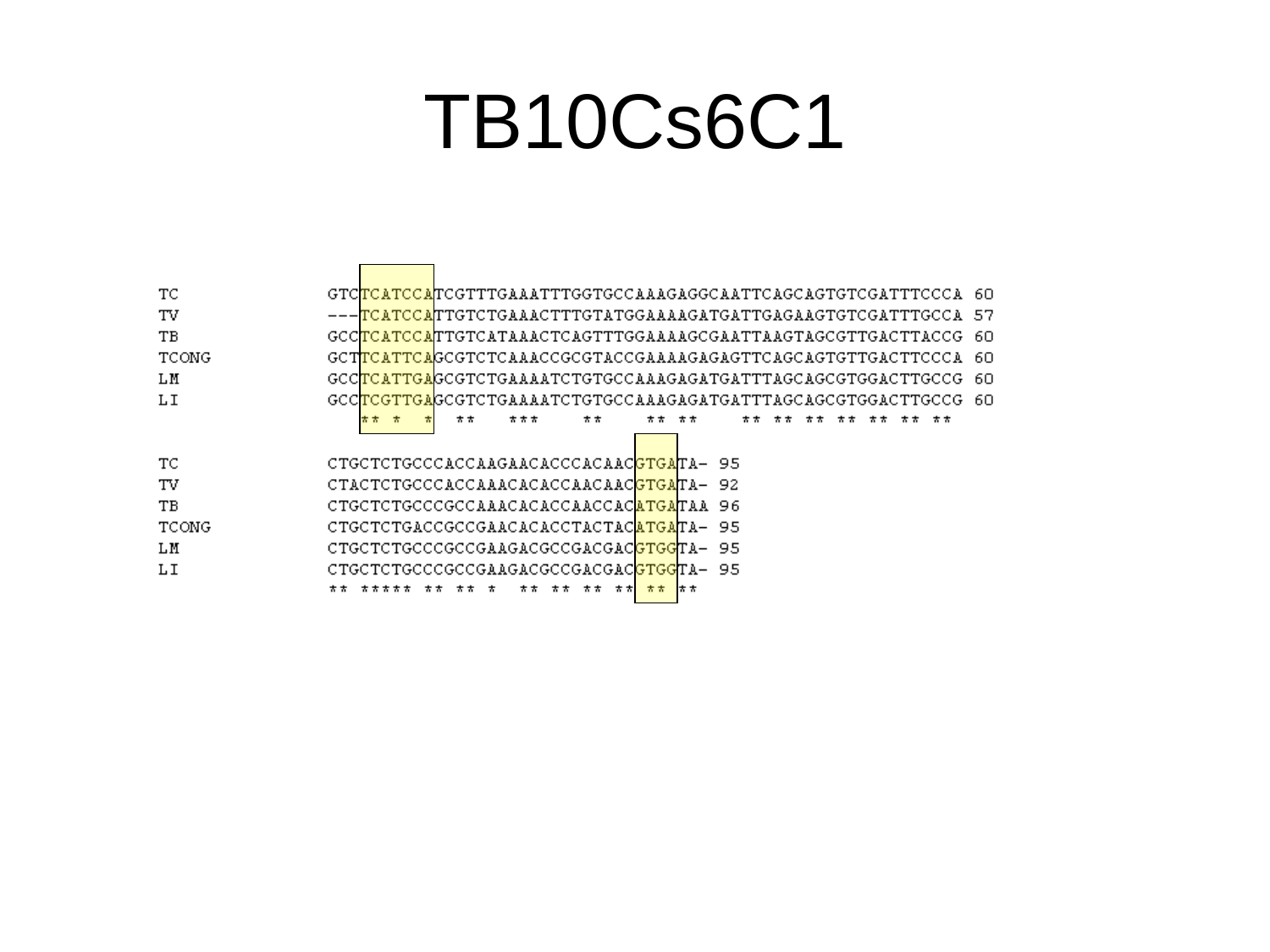

# TB10Cs6C1

## Slide 4
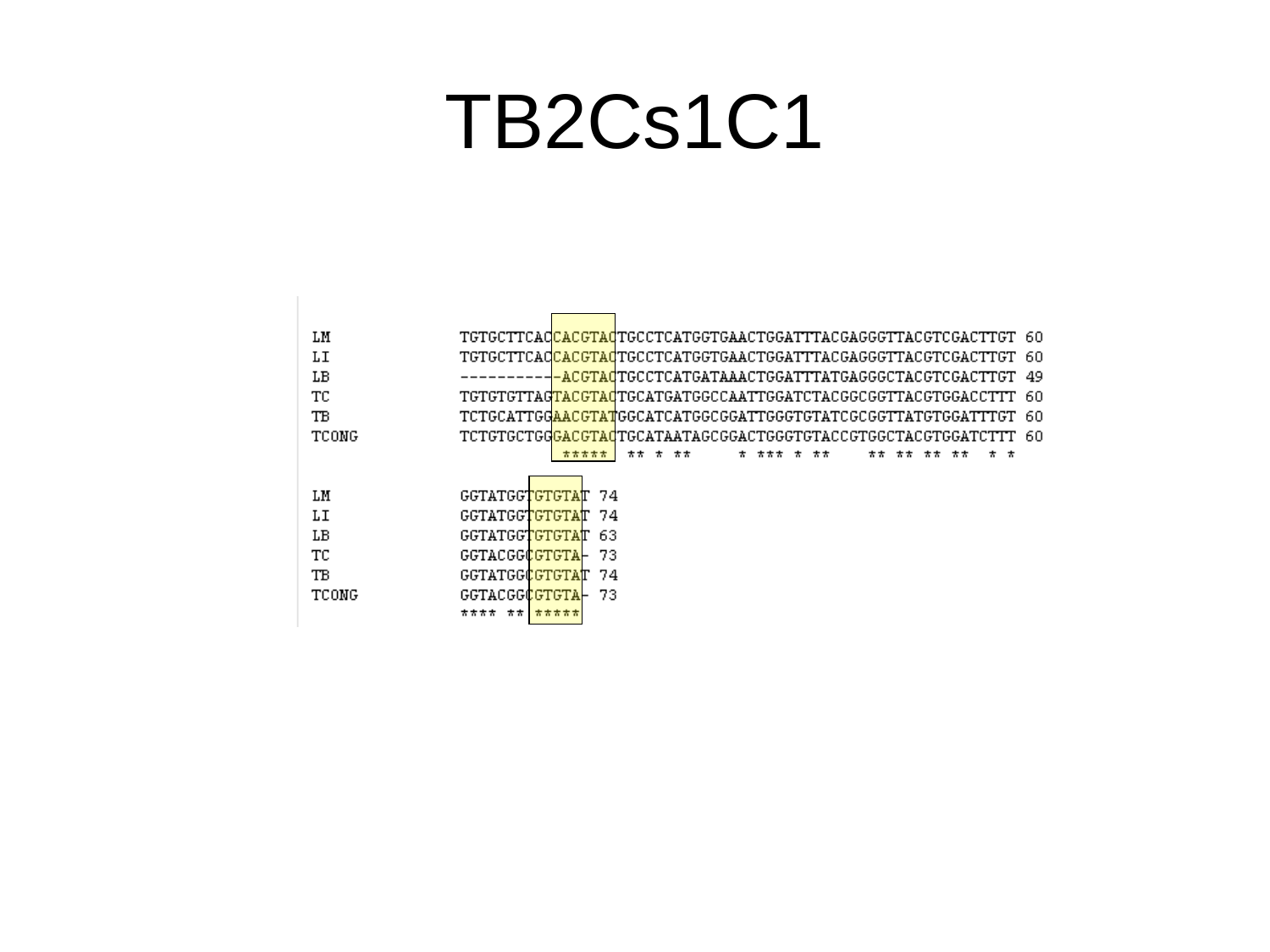

# TB2Cs1C1

Supplement: Additional file 7 — The box structure of the novel C/D molecules. For C/D snoRNA TB10Cs2"C3, TB10Cs2"C4, TB10Cs6C1, TB2Cs1C1 the canonical C and D box structure is shown. [file 1471-2164-11-615-S7.PPT]

## Slide 1
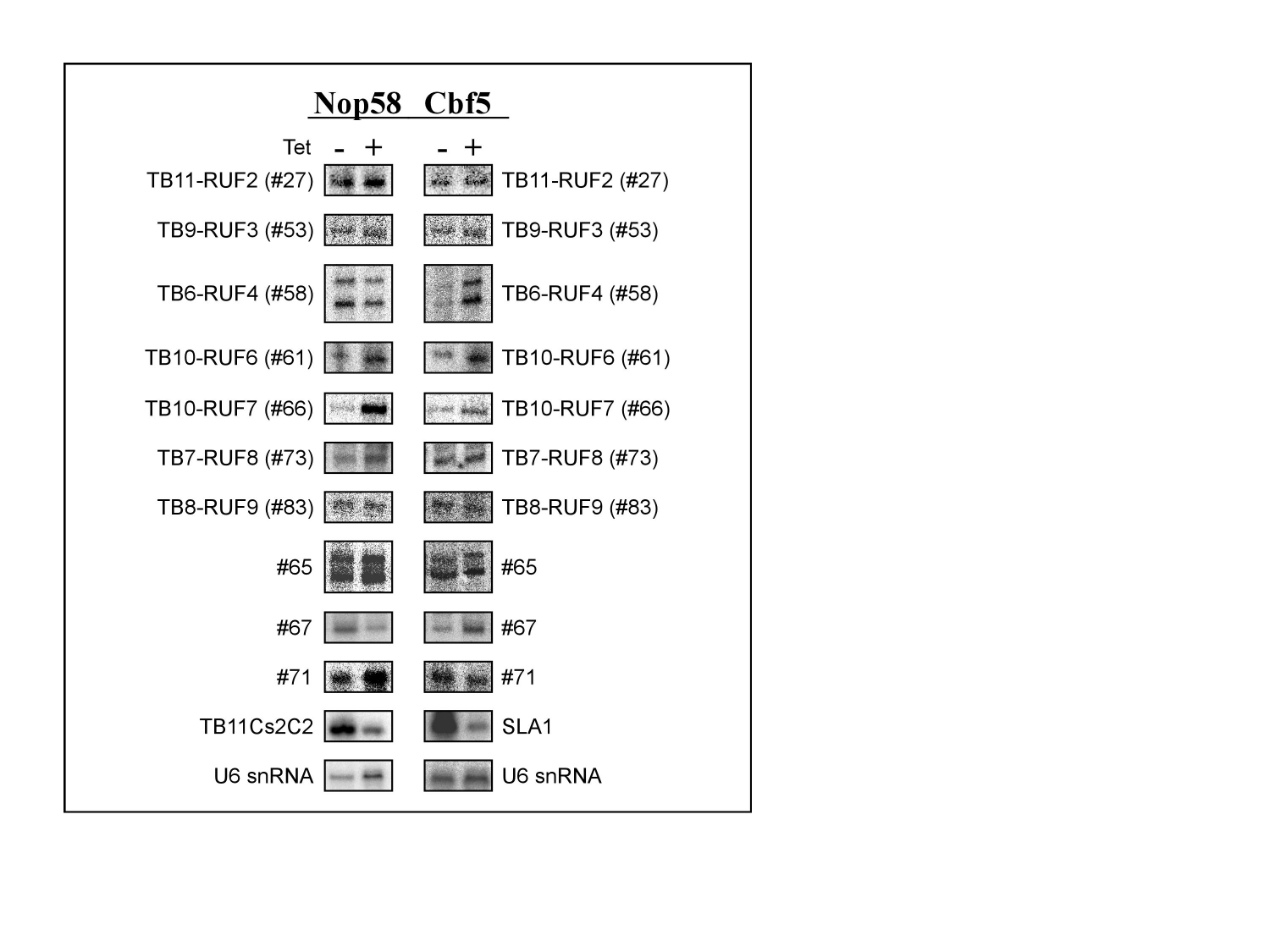

Supplement: Additional file 8 — Effects of NOP58 and CBF5 knockdown on all candidates. RNA was prepared from cells carrying either NOP58 or CBF5 silencing constructs before induction with Tetracycline (-) and 3 days after addition of tetracycline (+). The RNA was analyzed by primer extension and separated on a 6% denaturing polyacrylamide gel. The level of U6 snRNA was used to examine the amount of RNA the samples. The same RNA was used for the different primer extension assays. [file 1471-2164-11-615-S8.PPT]
