# Supplementary material for: A comparative genome-wide study of ncRNAs in trypanosomatids
Source: BMC Genomics. 2010 Nov 4;11:615. doi: 10.1186/1471-2164-11-615 (PMC3091756; doi:10.1186/1471-2164-11-615)
Supplement: Additional file 9 — GeneDB accession numbers of the new snoRNA molecules that were reported in this study. List of the newly annotated sequences with their GeneDB id. [file 1471-2164-11-615-S9.DOC]

Sequence data from this article have been deposited with the GeneDB with the following accession numbers:

Name in manuscript GeneDB Accession Number

TB2Cs1C1 Tb927_02_v4.snoRNA.0100

TB10Cs6C1 Tb927_10_v5.snoRNA.0108

TB10Cs2"C1 Tb927_10_v5.snoRNA.0100

TB10Cs2"C2 Tb927_10_v5.snoRNA.0101

TB10Cs2"C3 Tb927_10_v5.snoRNA.0102

TB10Cs2"C4 Tb927_10_v5.snoRNA.0103

TB10Cs2"C1 Tb927_10_v5.snoRNA.0104

TB10Cs2"C2 Tb927_10_v5.snoRNA.0105

TB10Cs2"C3 Tb927_10_v5.snoRNA.0106

TB10Cs2"C4 Tb927_10_v5.snoRNA.0107
